# Manipulating mtDNA *in vivo* reprograms metabolism via novel response mechanisms

**DOI:** 10.1371/journal.pgen.1008410

**Published:** 2019-10-04

**Authors:** Diana Bahhir, Cagri Yalgin, Liina Ots, Sampsa Järvinen, Jack George, Alba Naudí, Tatjana Krama, Indrikis Krams, Mairi Tamm, Ana Andjelković, Eric Dufour, Jose M. González de Cózar, Mike Gerards, Mikael Parhiala, Reinald Pamplona, Howard T. Jacobs, Priit Jõers

**Affiliations:** 1 Institute of Molecular and Cell Biology, University of Tartu, Tartu, Estonia; 2 Institute of Biotechnology, University of Helsinki, Helsinki, Finland; 3 Faculty of Medicine and Health Technology, Tampere University, Tampere, Finland; 4 Experimental Medicine Department, University of Lleida-Institute for Research in Biomedicine of Lleida (UdL-IRBLLEIDA), Lleida, Spain; 5 Institute of Ecology and Earth Sciences, University of Tartu, Tartu, Estonia; 6 Department of Plant Health, Institute of Agricultural and Environmental Sciences, Estonian University of Life Science, Tartu, Estonia; 7 Department of Zoology and Animal Ecology, Faculty of Biology, University of Latvia, Rīga, Latvia; 8 Department of Biotechnology, Daugavpils University, Daugavpils, Latvia; 9 Maastricht Centre for Systems Biology (MaCSBio), Maastricht University, Maastricht, The Netherlands; University of Cologne, GERMANY

## Abstract

Mitochondria have been increasingly recognized as a central regulatory nexus for multiple metabolic pathways, in addition to ATP production via oxidative phosphorylation (OXPHOS). Here we show that inducing mitochondrial DNA (mtDNA) stress in *Drosophila* using a mitochondrially-targeted Type I restriction endonuclease (mtEcoBI) results in unexpected metabolic reprogramming in adult flies, distinct from effects on OXPHOS. Carbohydrate utilization was repressed, with catabolism shifted towards lipid oxidation, accompanied by elevated serine synthesis. Cleavage and translocation, the two modes of mtEcoBI action, repressed carbohydrate rmetabolism via two different mechanisms. DNA cleavage activity induced a type II diabetes-like phenotype involving deactivation of Akt kinase and inhibition of pyruvate dehydrogenase, whilst translocation decreased post-translational protein acetylation by cytonuclear depletion of acetyl-CoA (AcCoA). The associated decrease in the concentrations of ketogenic amino acids also produced downstream effects on physiology and behavior, attributable to decreased neurotransmitter levels. We thus provide evidence for novel signaling pathways connecting mtDNA to metabolism, distinct from its role in supporting OXPHOS.

## Author summary

Mitochondria, subcellular compartments (organelles) found in virtually all eukaryotes, contain DNA which is believed to be a remnant of an ancestral bacterial genome. They are best known for the synthesis of the universal energy carrier ATP, but also serve as the hub of various metabolic and signalling pathways. We report here that mtDNA integrity is linked to a signaling system that influences metabolic fuel selection between fats and sugars. By disrupting mtDNA in the fruit fly we induced a strong shift towards lipid catabolism. This was caused both by a widespread decrease in post-translational acetylation of proteins, as well as specific inhibition of the machinery that transports glucose into cells across the plasma membrane. This phenomenon is very similar to the pathophysiology of diabetes, where the inability to transport glucose to cells is considered the main hallmark of the disease. Moreover, decreased protein acetylation was associated with lower levels of certain neurotransmitters, causing various effects on feeding and fertility. Our discovery reveals an unexpected role for mtDNA stability in regulating global metabolic balance and suggests that it could be instrumental in pandemic metabolic disorders such as diabetes and obesity.

## Introduction

Mitochondria have diverse roles in cellular metabolism: hosting the TCA cycle, controlling Ca^2+^ signaling, synthesizing FeS clusters and inducing cell death to name but a few. However, their best known role is to generate ATP via oxidative phosphorylation (OXPHOS), which is usually powered by two main substrate classes: carbohydrates and lipids. The selection of the fuel source for ATP generation is a dynamic multi-step process that can be rearranged to meet organismal needs. In addition to nuclear-encoded components of the electron transport chain (ETC), the process of mitochondrial ATP production requires a small number of crucial subunits encoded by mitochondrial DNA (mtDNA). The classic view of mtDNA disorders is that a lack of such subunits, or the presence of damaged subunits, leads to dysfunctional ETC complexes, which in turn causes pathological changes due to decreased ATP and increased production of damaging reactive oxygen species (ROS).

This paradigm is challenged by the large heterogeneity of pathological manifestations of mtDNA alterations that are unlikely to be caused by OXPHOS dysfunction alone [1]. An explanation can be provided by the various forms of mitochondrial communication with other cellular compartements [2–4]. Although mitochondrial biogenesis is under nuclear control, a number of retrograde pathways link mitochondrial homeostasis with other cellular functions. Their effects vary, ranging from the elimination of dysfunctional mitochondria by mitophagy and the induction of apoptosis, to nonlethal shifts in metabolism. Certain specific changes in mtDNA function, for example, mutations in tRNA genes, can trigger distinct stress responses linked to arrested translation and imbalance between nuclear- and mitochondrially encoded ETC subunits. While several of them may eventually lead to OXPHOS defects, there is a growing view that these stress signals could be primary contributors to pathology, in at least some mitochondrial disorders. Activating transcription factors (ATFs) are proposed to mediate this regulation, launching programs aimed at re-establishing homeostasis that have been described as the mitochondrial unfolded protein response (mtUPR) and the integrated stress response (ISR) [1[. Responses to mtDNA stress can be non-cell autonomous, such as via the systemic action of fibroblast growth factor 21 (FGF21) [5, 6]. Little is known about how these signaling pathways are activated, although the role of TCA intermediates as second messengers has been increasingly recognized [7], with effects on nucleic acid and protein modifications, such as methylation and acetylation [8, 9].

mtDNA is organized in nucleoids, protein-DNA complexes that are considered to be units of inheritance [10]. As well as factors for mtDNA transactions, they have been found to contain a number of proteins linked to metabolism, although their functions in the nucleoid are generally not known [11]. A prominent component of nucleoids is the DNA-packaging factor TFAM, that has a clear preference for negatively supercoiled DNA [12–14]. TFAM depletion can induce mitochondrial stress responses [15, 16] that have hitherto been consideres to be mediated by OXPHOS impairment [17]. Similar arguments can be applied to the „deletor”mouse model, where due to defective DNA helicase mtDNA deletions accumulate at a slow rate, leading to late-onset mitochondrial dysfunction [18]. The mice manifest metabolic alterations [19, 20] that are assumed to be OXPHOS related, although other types of signaling may be involved.

Here we provide evidence for a previously unkown role of mtDNA stability in reprogramming the use of metabolic pathways. We manipulated mtDNA *in vivo* by targeting a bacterial Type I restriction endonuclease (RE), EcoBI, to *Drosophila* mitochondria. These complex enzymes are capable of both DNA cleavage and methylation depending on the methylation states of their target sequences [21]. In fact, partial complex consisting of only two subunits (HsdM and HsdS) is still capable of methylating target DNA. After binding to their target sequences, Type I REs translocate along DNA before introducing double-strand breaks (DSBs), causing essentially random cleaveage [21]. When induced early in development, we found that mitochondrial EcoBI (mtEcoBI) disrupted mtDNA as expected, through cleavage and topological aberrations resulting from translocation activity. This led to severe ETC defects and increased ROS production, with expected downstream effects on cellular homeostasis and systematic lethality. However, mtDNA damage by adult-onset expression of mtEcoBI was much more limited and did not interfere with OXPHOS. Nevertheless, it led to major metabolic alterations, resulting in lethality within two weeks. This effect was brought about by two distinct mechanisms: translocation activity decreased cytonuclear protein acetylation due to lower cytosolic AcCoA, whilst cleavage of mtDNA inactivated the Akt kinase. Both effects converged in a multifaceted inhibition of carbohydrate catabolism, causing a shift towards lipid oxidation. Furthermore, serine synthesis was increased, a phenomenon observed also in other mtDNA stress conditions [20, 22, 23]. Finally, depletion of ketogenic amino acids capable of replenishing cytosolic AcCoA caused strong effects on feeding and fertility via decreased levels of tyrosine-derived neurotransmitters. These findings represent the first clear-cut demonstration of the activation of a metabolic stress-response pathway by mtDNA disruption, independently of any measurable disturbance of OXPHOS.

## Results

### Early-onset expression of mtEcoBI induces larval lethality and mitochondrial dysfunction

**Fig 1:**
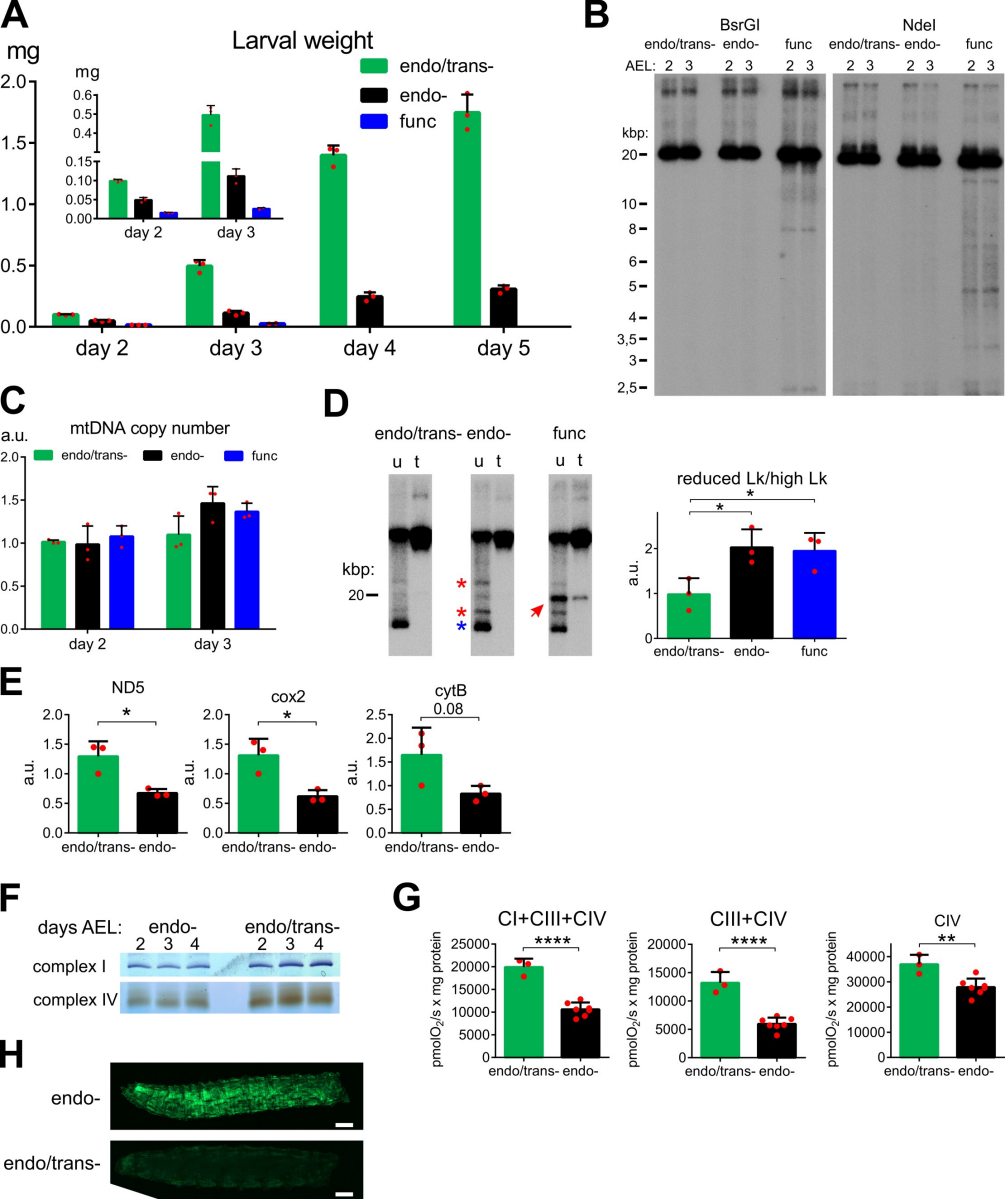
Phenotype of early-onset expression of mtEcoBI isoforms. **(A)** Wet weight of larvae of strains expressing *daGAL4*-driven mtEcoBI isoforms (*UAS-mtHsdM*.*UAS-mtHsdS*/+;*UAS-mtHsdR* K477R/*daGAL4* (endo/trans-), *UAS-mtHsdM*.*UAS-mtHsdS*/+;*UAS-mtHsdR* D298E/*daGAL4* (endo-), *UAS-mtHsdM*.*UAS-mtHsdS*/+;*UAS-mtHsdR*/*daGAL4* (func)) from days 2 to 5 after egg laying (AEL), n = 3. Inset graph shows weight for first two days. **(B)** BsrGI- and NdeI-cleaved mtDNA from the same strains isolated 2 and 3 days AEL. (**C)** mtDNA copy number for all three strains measured at 2 and 3 AEL. **(D)** Topology of uncut mtDNA treated (t) or untreated (u) with topoisomerase I from the same strains isolated 3 days AEL and quantification of circular isoforms with reduced linking number (Lk), *p*<0.05 (*). Red arrow indicates the linear forms and red asterisks mark circular forms with lower negative supercoiling than the major closed circular form (blue asterisk). **(E)** Steady-state transcript levels of mitochondrial genes ND5 (*mt*:*ND5*), cox2 (*mt*:*CoII*) and cytB (*mt*:*Cyt-b*) in larvae of these strains at day 5 AEL, *p*<0.05 (*), n = 3. **(F)** In-gel activity of ETC complexes I and IV isolated from larvae expressing *da*GAL4-driven mtEcoBI isoforms as indicated, at days 2, 3 and 4 AEL. **(G)** State III respiration of mitochondria isolated from larvae of the indicated strains at comparable developmental stage (days 2 and 3 AEL respectively, n = 3 for day 2 and n = 7 for day 3 larvae), *p*<0.01 (**), *p*<0.0001 (****). **(H)** Microscopy of larvae from strains *UAS-mtHsdM*.*UAS-mtHsdS*/*tub-Orp1GFP*; *UAS-mtHsdR* K477R/*daGAL4* and *UAS-mtHsdM*.*UAS-mtHsdS*/ *tub-Orp1GFP*; *UAS-mtHsdR* D298E/*daGAL4* with identical recording parameters. Scale bar is 0.2 mm.

Type I REs such as EcoBI are heterotrimeric enzymes. To target EcoBI to the mitochondrial matrix in *Drosophila* we therefore fused the coding sequences for each of its three subunits (HsdM, HsdS and HsdR) to the robust mitochondrial targeting sequence from the citrate synthase gene [24] and placed them under the control of GAL4-dependent UAS elements. Expression of each subunit was confirmed by qRT-PCR in flies and mitochondrial localization by immunocytochemistry in S2 cells and by western blots of subcellular protein extracts from *Drosophila* tissue (S1A–S1C Fig). We generated three isoforms for the HsdR (endonuclease) subunit: a fully functional wild-type version (func) and two others, each with single point-mutations either in the endonuclease (D298E) or ATPase (K477R) domain, rendering the subunit respectively deficient in endonuclease (endo-) and both endonuclease and translocation (endo/trans-) functions [25]. These mutations did not alter the stability of the protein, as its abundance remained unchanged compared to the func isoform (S1D Fig). Ubiquitous co-expression of the mitochondrially targeted HsdM and HsdS subunits, together with the endo/trans- HsdR isoform, using the *daughterless* GAL4 (*daGAL4*) driver, had no effect on development or viability (Fig 1A). In contrast, co-expression of the func HsdR isoform resulted in decreased larval wet weight and early larval lethality at L1/L2 stage (Fig 1A). The endo- isoform also produced larval lethality but with increased survival into L3 stage, nevertheless with severely decreased weight gain. MtDNA integrity was detectably affected only in the func strain, with random shearing combined with specific cleavage sites near the replication origin, the NCR border and also at the two binding sites for mTTF/mTERF5 (Fig 1B and S2A Fig). This is consistent with the known proclivity of Type I REs to cleave DNA when they encounter a bound protein or higher-order DNA structure inferred to be present in those mtDNA regions [26]. The results indicate fragmention of mtDNA by mtEcoBI, without a decrease in copy number (Fig 1C) or modification of its binding sequence (S2B Fig). Translocation also resulted in a shift towards circular isoforms with decreased linking number, seen for both the endo- and func strains (red asterisks in Fig 1D and S2C Fig). In accordance with the expectation that this topological disturbance would affect gene expression (as described in human cells [27]), we observed that steady-state levels of mtDNA transcripts were decreased in larvae expressing the endo- enzyme (Fig 1E). An effect on the production of mitochondrially encoded proteins was consistent with decreased in-gel activity of OXPHOS complexes I and IV (Fig 1F) and diminished respiratory chain activity both in larvae of similar size or same chronological age (Figs 1G and S2D), independent of the substrate used. These defects in respiration were accompanied by elevated ROS production as demonstrated by increased fluorescence of an *in vivo* GFP-based ROS marker (Fig 1H). Increased ROS contributed to the lethal phenotype, since the co-expression of mitochondrial catalase (mCat) and superoxide dismutase 2 (SOD2) alleviated the developmental defect while GFP did not (S2E Fig). Additional evidence of elevated ROS due to mtEcoBI action comes from the overproliferation of lamellocytes (S3A Fig), type of immunological cell that can be induced by elevated ROS production [28]. Lamellocyte proliferation leads to the formation of melanotic nodules, as observed in endo- larvae, but was suppressed by SOD2 overexpression or by feeding larvae ROS scavenger N-acetylcysteine (S3B Fig).

### Adult-onset expression of mtEcoBI causes lethality without mitochondrial OXPHOS deficiency

**Fig 2:**
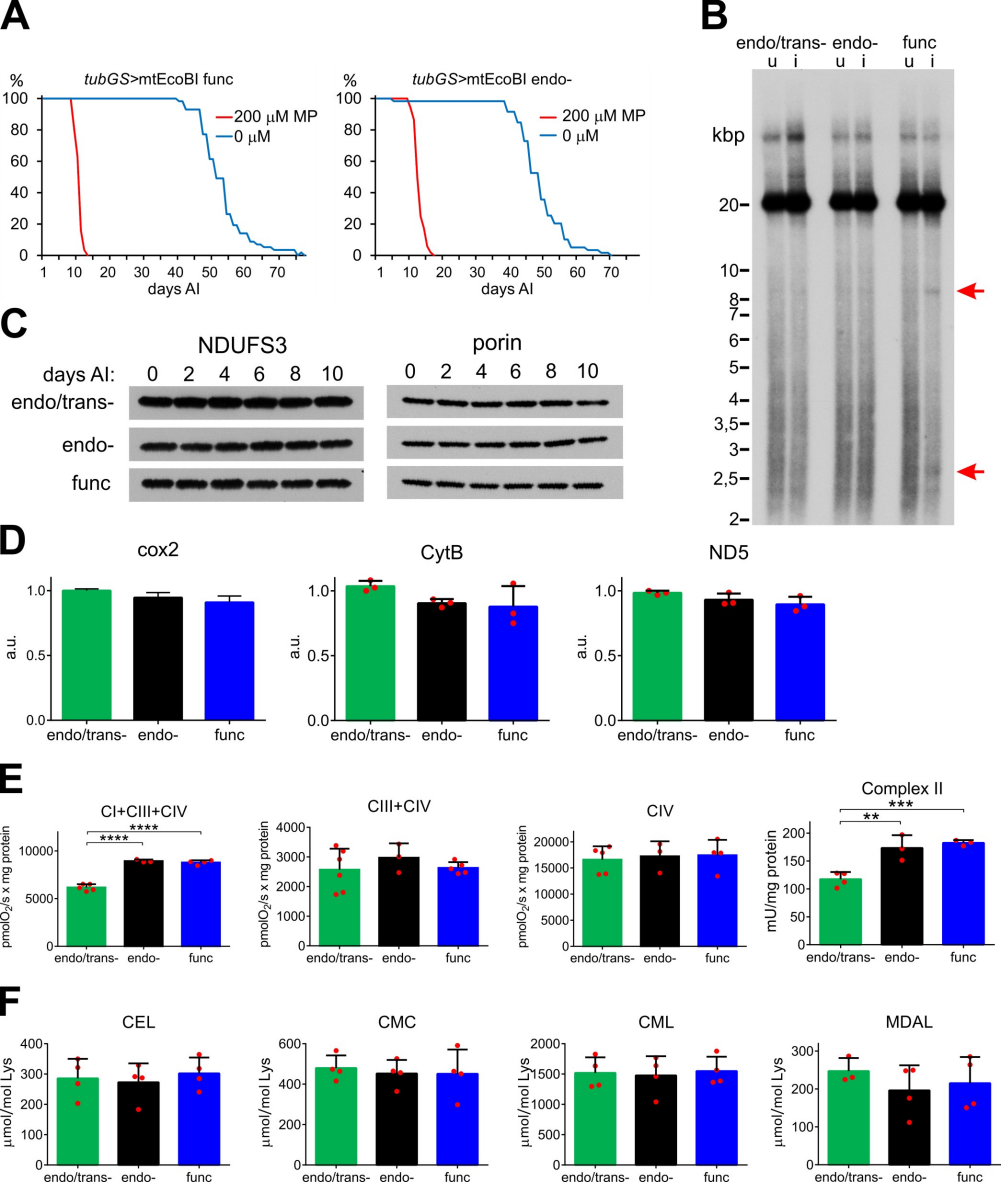
Phenotype of adult-onset expression of mtEcoBI isoforms driven by *tubGS*. **(A)** Lifespans of *tubGS*>mtEcoBI func (*UAS-mtHsdM*.*UAS-mtHsdS*/+;*UAS-mtHsdR*/*tubGS*) and endo- (*UAS-mtHsdM*.*UAS-mtHsdS*/+;*UAS-mtHsdR* D298E/*tubGS*) strains after induction (AI) on 200 μM mifepristone (MP). **(B)** Topology of BsrGI-digested mtDNA from *tubGS*>mtEcoBI flies, u-uninduced, i-induced for 10 days on 200 μM MP-containing food. Red arrows indicate predicted products from cleavage at mTTF/mTERF5 binding sites. **(C)** Western analysis of mitochondrial proteins NDUFS3 (ND-30) and porin in *tubGS*>mtEcoBI strains from days 0 to 10 AI with 200 μM MP (see S5 Fig. for normalization control). **(D)** Steady-state transcripts levels of cox2, cytB and ND5 in *tubGS*>mtEcoBI strains 10 days after induction AI with 200 μM MP, n = 3. **(E)** Activities of ETC chain complexes in either state III respiration of mitochondria (cI, cIII and cIV, n = 3–6) or according to formation of product (cII, n = 3–4) in *tubGS*>mtEcoBI strains 6 days AI with 200 μM MP. *p*<0.01 (**), *p*<0.001(***), *p*<0.0001(****). **(F)** Protein carbonylation levels in *tubGS*>mtEcoBI strains at 10 days after induction (AI) with 200 uM MP. CEL-carboxyethyl-lysine, CML-carboxymethyl-lysine, CMC-carboxymethyl-cysteine, MDAL-malondialdehyde-lysine, n = 4.

Since the effects of mtEcoBI expression were lethal at larval stages, we used the mifepristone (MP)-inducible tubulin GeneSwitch driver (*tubGS*) to induce the expression of mtEcoBI and determine its phenotypic effects in the adult fly. Expression of the func or endo- isoforms again resulted in lethality, following 10 days of induction (Fig 2A), while no adverse effects were seen in the strain expressing the endo/trans- isoform, nor that expressing only the partially active methyltransferase complex (MTase) consisting of the HsdM and HsdS subunit or those expressing these subunits individually (S4A and S4B Fig). Both the development and lifespan of the induced *tubGS*>mtEcoBI endo/trans- strain were broadly similar to those of the *w*^*1118*^ parental strain (S4A and S4C Fig). Lethality was preceded by the onset of serious locomotor dysfunction on day 7 (S4D Fig). The extent of degradation of mtDNA just before death was less than that seen in larvae (Fig 2B) even though the same preferred cleavage sites at the mTTF/mTERF5 binding sites were detected. As during larval stage, no modification of mtEcoBI’s binding site could be detected (S4E Fig). We tested whether mtEcoBI was specifically targeting mtDNA molecules undergoing replication, since DNA synthesis requires local unwinding that might be disrupted by mtEcoBI. However, two-dimensional neutral gel electrophoresis (2DNAGE) showed qualitatively and quantitavely normal replication intermediates (RIs) (S5 Fig). Consistent with this more limited amount of damage to mtDNA, no significant alteration in mitochondrial transcript or protein levels was observed (Fig 2C and 2D and S6 Fig). State III respiration indicated no ETC defect, while coupled respiration from complex I-linked substrates was in fact increased in the endo- and func mtEcoBI strains, as was the activity of complex II (Fig 2E). Quantification of various forms of oxidative damage to proteins (considered as markers for elevated ROS) showed no increase compared with controls, even after 10 days of induction (Fig 2F). Furthermore, fly brains dissected at day 6 and stained with dihydroethidium (DHE), a sensitive dye for detecting superoxide *in vivo*, showed no evidence for any increase in ROS (S7A Fig). Overexpression of ROS scavengers SOD2 and mCat in endo- and func mtEcoBI expressing flies did not modify the lethal phenotype (S7B Fig), in contrast to their alleviating effect in larvae. Together these results imply that the lethal adult phenotype is not mediated by increased ROS or OXPHOS deficiency.

Although steady-state mitochondrial transcript levels were the same as in control flies, qRT-PCR does not reflect RNA integrity. Accumulation of truncated mRNAs could lead to the formation of aberrant proteins that would trigger mtUPR. We therefore analyzed mRNA and protein levels for a number of chaperones and proteases known to be upregulated when the mtUPR is induced. None of these markers showed any upregulation (S8A Fig). Similarly, the phosphorylation status of eIF2α was unaltered, consistent with no activation of the integrated stress response (ISR) (S8B Fig).

### In adults mtEcoBI induces metabolic reprogramming

**Fig 3:**
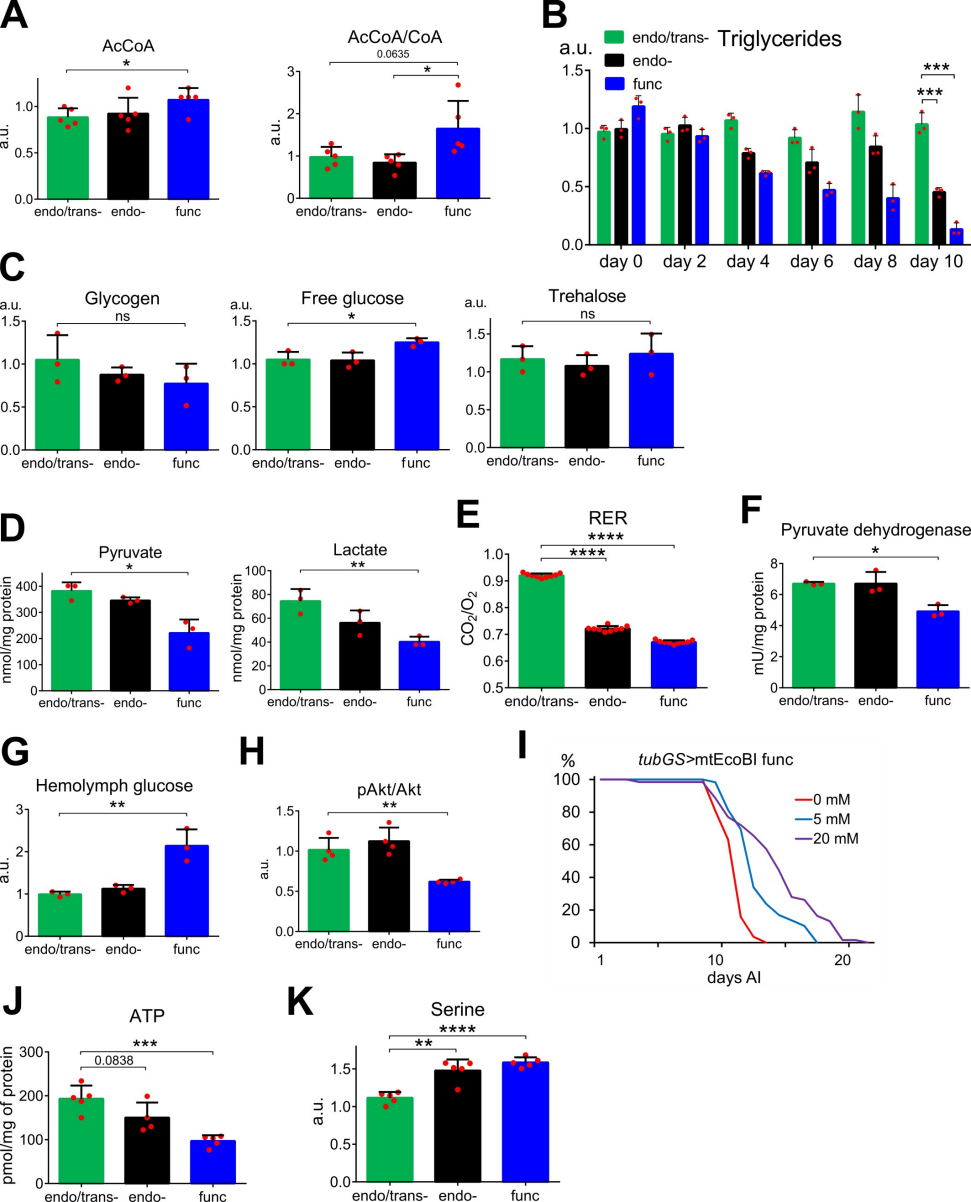
Metabolite levels and insulin pathway activity during adult-onset systemic mtEcoBI expression. **(A)** AcCoA and AcCoA/CoA ratio in *tubGS*>mtEcoBI flies 6 days after induction (AI) with 200 μM MP, *p*<0.05 (*), n = 5. **(B)** Relative TAG (triglyceride) levels normalized to protein content between days 0 and 10 AI with 200 μM MP, in *tubGS*>mtEcoBI strains, *p*<0.001 (***), n = 3. **(C)** Levels of carbohydrates (normalized to protein content) in *tubGS*>mtEcoBI strains 10 days AI with 200 μM MP, *p*<0.05 (*), ns–not significant, n = 3. **(D)** Pyruvate and lactate concentrations (normalized to protein content) in *tubGS*>mtEcoBI strains 6 days AI, *p*<0.05 (*), *p*<0.01 (**), n = 3. **(E)** RER measurements in *tubGS*>mtEcoBI strains 6 days AI with 200 μM MP, *p*<0.0001(****), n = 9. **(F)** Pyruvate dehydrogenase activity in *tubGS*>mtEcoBI strains 6 days AI, *p*<0.05 (*), *p*<0.01 (**), n = 3. **(G)** Hemolymph glucose levels in *tubGS*>mtEcoBI strains, 0 and 10 days AI with 200 μM MP, *p*<0.05 (*), n = 3. **(H)** Ratio of phospho-Akt to pan-Akt in *tubGS*>mtEcoBI strains at day 10 AI with 200 μM MP, *p*<0.01 (**), n = 4. **(I)** Lifespan of *tubGS*> mtEcoBI func strain on food with 200 μM MP with either 0mM, 5 mM or 20 mM metformin (respective *p*-values: 1.5 x 10^−8^ for 5 mM vs. control and 9.7 x 10^−9^ for 20 mM vs. control). Lifespan on food without metformin is a replicate from Fig 2A to provide a better comparison with metformin effect. **(J)** ATP levels in *tubGS*>mtEcoBI strains 6 days AI with 200 μM MP, *p*<0.001(***), n = 5. **(K)** Serine levels in *tubGS*>mtEcoBI strains 6 days AI with 200 μM MP, *p*<0.01 (**), *p*<0.001(***), n = 5.

The lethality of mtEcoBI expression, despite mitochondrial respiration remaining functional, prompted us to analyze changes in metabolic footprint. Metabolomic analysis detected an accumulation of TCA cycle intermediates along with the odd-chain fatty acid oxidation product propanoyl-CoA and elevated AcCoA, as well as an increased AcCoA/CoA ratio (Fig 3A and S9 Fig). This suggested an increased reliance on lipid oxidation to fuel mitochondria. Triacylglycerides (TAG), a major class of energy storage molecule in *Drosophila*, were progressively depleted starting already at day 4 after mtEcoBI induction and were decreased by 90% in the func mtEcoBI-expressing flies just before death (Fig 3B). The depletion was less dramatic for the endo- strain, which also had a slightly longer lifespan. No such depletion was seen in the *w*^*1118*^ control strain, nor in flies expressing the MTase-competent combination of HsdM and HsdS (S10A Fig). At the same time, total glucose was slightly elevated in the func strain and there were no statistically significant changes in the levels of glycogen and trehalose, two major carbohydrate storage molecules (Fig 3C and S10B Fig), whilst the glycolytic end-products pyruvate and lactate were decreased (Fig 3D). Consistent with a shift in catabolic fuel source, we observed a decrease in the respiratory exchange ratio (RER) to 0.7, indicative of complete reliance on triglycerides for energy (Fig 3E). This was accompanied by lower pyruvate dehydrogenase activity in the func mtEcoBI strain (Fig 3F). Importantly, we observed a strong elevation of hemolymph glucose in the func mtEcoBI strain, accompanied by a marked decrease in phosphorylation of Akt kinase (Fig 3G–3H and S10C Fig). Addition of the antidiabetic drug metformin, which promotes glucose uptake in *Drosophila* tissues [29], delayed the lethal effect of mtEcoBI expression in the func but not in the endo- strain, nor did it alter the survival of endo/trans- or *w*^*1118*^ parental strain flies (Fig 3I and S10D Fig). These metabolic changes were associated with the suppression of insulin signaling, indicated by the induction of the markers for insulin pathway inhibition InR, ImpL3 (Ldh) and 4E-BP (S10E Fig) [30].

To address the tissue-specificity of the observed phenotype, we induced the expression of the mtEcoBI variants in muscle, a major catabolic tissue. The phenotype was essentially identical to that of *tubGS>*mtEcoBI flies: in the strain expressing func mtEcoBI in muscle, again with adult onset, we observed lethality with the same timing as with ubiquitous expression, accompanied by only limited mtDNA cleavage, no defect in OXPHOS but decreased triacylglyceride levels and elevated hemolymph glucose (S11 Fig).

The *tubGS>*mtEcoBI flies also showed decreased steady-state levels of ATP (Fig 3J and S12A Fig). Despite the evidence indicating a block on carbohydrate usage, none of the three rate-limiting enzymes of glycolysis, phosphofructokinase (PFK), hexokinase (HX) and pyruvate kinase (PyK), demonstrated any decrease in activity (S12B Fig). In contrast, we noticed a 2-fold elevation in the levels of phosphoenolpyruvate (PEP) and 3-phosphoglycerate (3-PG), but not dihydroxyacetone phosphate (DHAP) or fructose 1,6-bisphosphate (FBP) in the func mtEcoBI strain (S13 Fig). 3-PG serves as a major branch point supplying serine synthesis, commonly elevated in response to mitochondrial dysfunction [23]. Accordingly, serine and its intermediate phosphoserine were elevated (Fig 3K and S13 Fig).

### mtEcoBI expression causes specific amino acid and neurotransmitter deficiency

**Fig 4:**
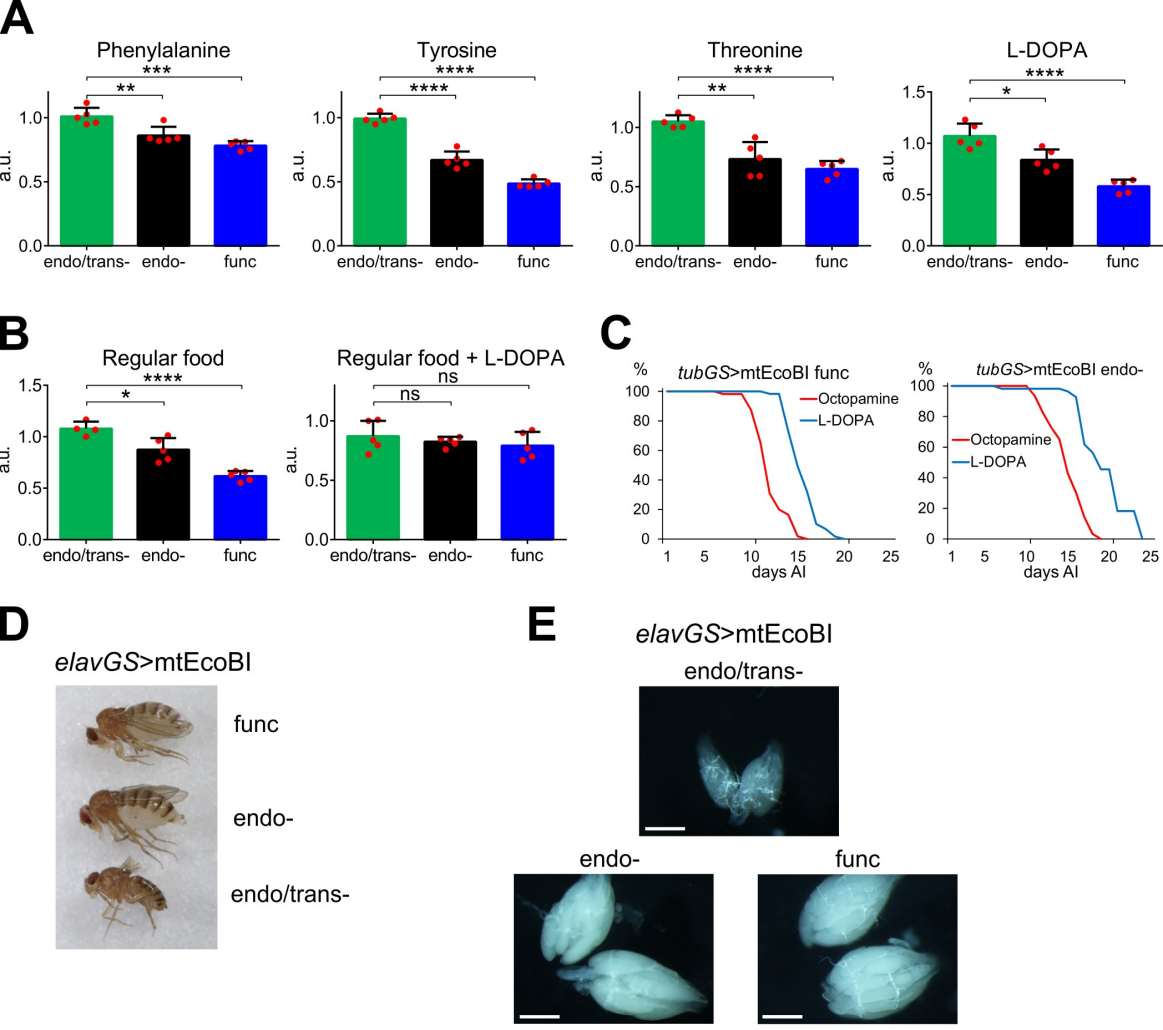
Neurotransmitter modulation during adult-onset mtEcoBI expression. **(A)** Relative levels of phenylalanine, tyrosine, threonine and L-DOPA in the indicated strains when driven by *tubGS*, 6 days after induction (AI) with 200 μM MP, *p*<0.05 (*), *p*<0.01 (**), *p*<0.001 (***), *p*<0.0001 (****), n = 5. **(B)** Relative food consumption of *tubGS*-driven mtEcoBI-expressing flies of the indicated strains either on regular or L-DOPA-supplemented food, 6 days AI with 200 μM MP, *p*<0.05 (*), *p*<0.0001 (****), ns–not significant, n = 4–5. **(C)** Lifespans of *tub*GS>mtEcoBI func and endo- strains maintained either on L-DOPA or octopamine-supplemented food. **(D)** Morphology of females from strains expressing mtEcoBI driven by *elavGS* 45 days after induction (AI) with 200 μM MP. **(E)** Dissected ovaries from aged females of strains expressing mtEcoBI driven by *elavGS*, 50 days AI on 200 μM MP, scale bars are 0.25 mm.

There was a clear decrease in the endo- and func strains in three ketogenic amino acids; phenylalanine, threonine and tyrosine (Fig 4A). Degradation of the first two produce fumarate, a TCA cycle intermediate. However, this did not apply to all ketogenic amino acids, and two anaplerotic amino acids, aspartate and glutamate, were elevated (S14 Fig). Tyrosine is also a known source of neurotransmitters, including dopamine. Its precursor, L-DOPA, demonstrated a similar decrease as tyrosine (Fig 4A). As dopamine regulates several aspects of *Drosophila* locomotion and behavior [31, 32], we investigated whether tyrosine deficiency was accompanied by any behavioral changes. Feeding intensity, which is controlled by dopamine, was lower in endo- and func mtEcoBI strains compared to the control, while L-DOPA complementation restored wild-type feeding behavior (Fig 4B). L-DOPA-supplemented food also displayed a limited rescue of lifespan, while another tyrosine-derived neurotransmitter, octopamine did not (Fig 4C). In addition, when mtEcoBI expression was induced specifically in adult neurons, using the *elavGS* driver, females showed severely bloated abdomens (Fig 4D). This was due to egg retention, as shown by the extensive accumulation of late-stage follicles in ovaries (Fig 4E). Notably, this phenotype is associated with deficiency of two other tyrosine-linked neutransmitters, octopamine and tyramine, which control egg deposition in *Drosophila* [33, 34].

### Effects of mtEcoBI expression are consistent with cytosolic AcCoA depletion

**Fig 5:**
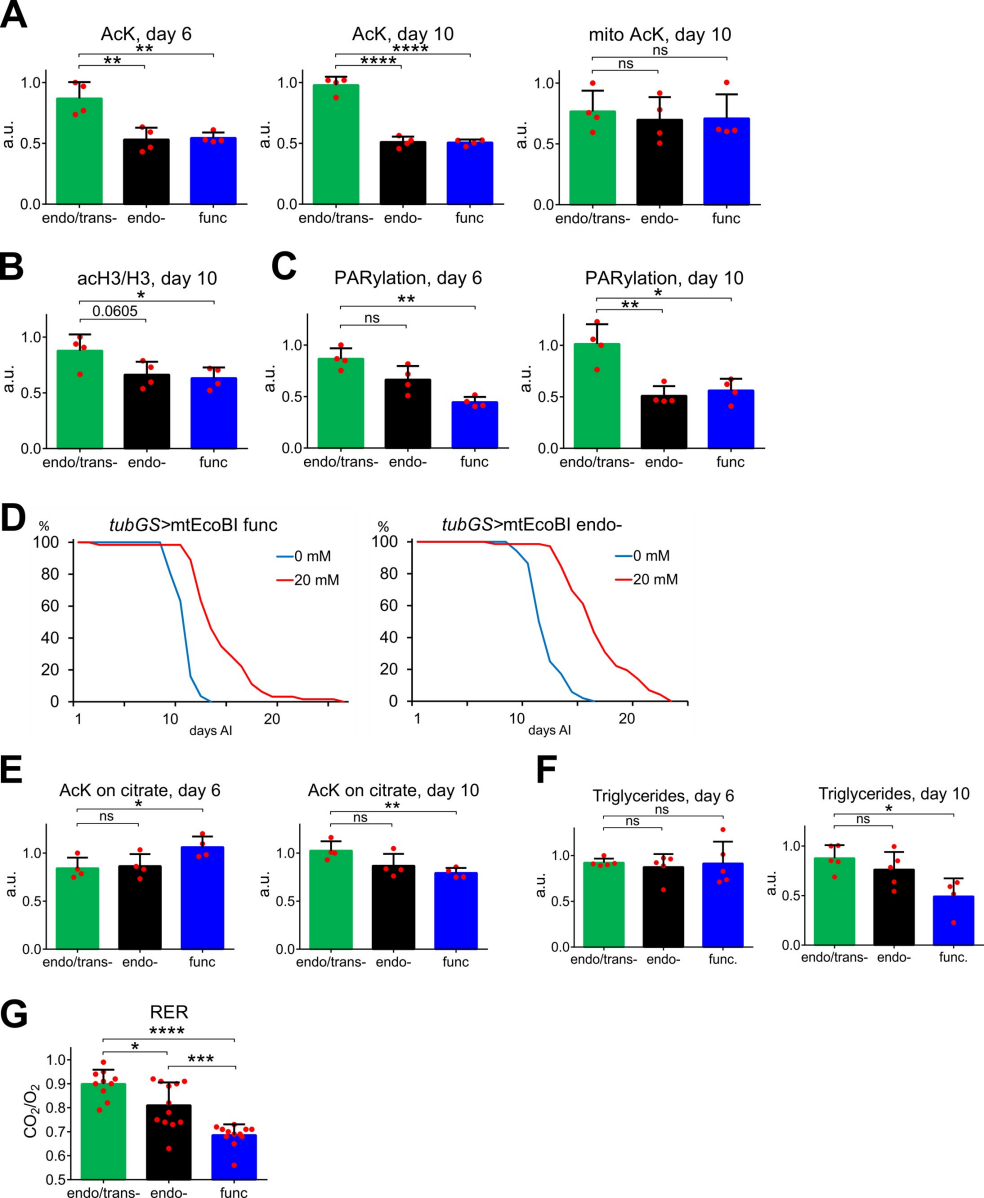
Post-translational modifications and effect of citrate on mtEcoBI-induced effects. **(A)** Relative levels of lysine acetylation (AcK) of total and mitochondrial proteins extracted from *tubGS*>mtEcoBI strains on days 6 and 10 after induction (AI) with 200 μM MP, *p*<0.01 (**), *p*<0.0001(****), n = 4. **(B)** Relative acetylation levels of histone H3 in *tubGS*>mtEcoBI strains 10 days AI with 200 μM MP (due to high variability two technical repeats were used). **(C)** Relative levels of poly-ADP-ribosylation of proteins in *tubGS*>mtEcoBI strains on days 6 and 10 AI with 200 μM MP *p*<0.05 (*), *p*<0.01 (**), n = 4. **(D)** Lifespans of *tubGS*>mtEcoBI endo- and func strains on food with 200 μM MP and with or without 20 mM sodium citrate. Lifespans on food without sodium citrate are replicates from Fig 2A to provide a better comparison with sodium citrate effects. **(E)** Total protein acetylation in *tubGS*>mtEcoBI strains on days 6 and 10 AI on food containing 200 μM MP and 20 mM sodium citrate, *p*<0.05 (*), *p*<0.01 (**), ns—not significant, n = 4. **(F).** Triglyceride levels in *tubGS*>mtEcoBI strains on days 6 and 10 AI on food containing 200 μM MP and 20 mM sodium citrate, *p*<0.05 (*), ns—not significant, n = 4–5. **(G)** RER of *tubGS*>mtEcoBI flies on day 6 after induction on food with 200 μM MP and 20 mM sodium citrate, *p*<0.05 (*), *p*<0.001(***), *p*<0.0001(****), n = 9–12.

Catabolism of tyrosine, phenylalanine and threonine (via threonine aldolase), unlike other ketogenic amino acids, produces AcCoA in the cytosol. Therefore, their deficiency suggests a resulting effect on cytosolic AcCoA. Outside of mitochondria, the donation of acetyl groups by this metabolite is required for *de novo* lipogenesis, the mevalonate pathway and protein acetylation. Total protein acetylation was indeed found to be significantly decreased on days 6 and 10, although mitochondrial proteins were unaffected (Fig 5A and S15A and S15B Fig). Analysis of the acetylation state of histone 3 demonstrated a similar decrease (Fig 5B and S15B Fig). Changes in acetylation were accompanied by a decrease of another post-translational modification, poly-(ADP) ribosylation, albeit with slower kinetics (Figs 5C and S15B). The concomitantly decreased NAD+/NADH ratio (S15C Fig) along with histone 3 acetylation defect implies that decreased protein modification is due to extramitochondrial AcCoA defciency, rather than the activation of NAD-dependent deacetylases. Providing flies with exogenous citrate, in order to replenish cytosolic AcCoA, led to a marked increase in lifespan in both endo- and func mtEcoBI strains (Fig 5D). This was accompanied by correction of the protein acetylation defect and a much slower loss of triacylglycerides (Fig 5E and 5F). Citrate-supplemented endo- mtEcoBI flies also had significantly elevated RER on day 6 compared with those maintained on regular food (Fig 5G and S16A Fig). Oxaloacetate-supplemented food did not provide any benefit (S16B Fig), excluding conversion to pyruvate (see S16C Fig) as a relevant mechanism. The findings support the idea that the metabolic changes are brought about by decreased cytonuclear protein acetylation due to cytosolic AcCoA depletion.

## Discussion

We have identified a novel, mtDNA stability-related pathway, unrelated to downstream OXPHOS defects, as a mediator of metabolic homeostasis in adult animals. This was discovered by using a novel approach to manipulate mtDNA *in vivo*, using a mitochondrially-targeted Type I restriction endonuclease.

Cleavage of mtDNA by mtEcoBI occurred preferentially at previously identified or inferred protein binding sites [32, 33], as typical for Type I restriction enzymes [34]. In accordance with reported effects of Type I enzymes [35], the translocation activity of mtEcoBI inflicts a significant torsional stress on target DNAs, disrupting their *in vivo* conformation, as reflected here by the enhancement of specific abnormal topoisomers. In larvae, this was accompanied by decreased levels of mitochondrial transcripts and OXPHOS impairment, similar to a previous report where mtDNA topology was disturbed pharmacologically [27]. This was sufficient to significantly impair growth and arrest development, leading ultimately to larval lethality with a quantitatively stronger effect if endonuclease activity was intact.

The effect on mtDNA was much weaker in adults, yet still led to early lethality, despite the lack of any detectable OXPHOS defect. Instead it caused an enforced switch to lipid catabolism with suppression of glycolysis resulting in rapid depletion of triglyceride, but not carbohydrate reserves. Strikingly, translocational activity of mtEcoBI alone was capable of decreasing acetylation of cytonuclear histone and nonhistone proteins. A reduced NAD^+^/NADH ratio pointed towards decreased cytosolic AcCoA levels as a reason for defective acetylation, further supported by decrease in endogenous sources of cytosolic AcCoA (Tyr, Phe, Thr). Inhibition of this common protein modification in the cytosol can have profound effects on cellular homeostasis through genome instability and induction of autophagy [36, 37]. Moreover, protein acetylation can affect other post-translational modifications, furthering its influence down multiple pathways. This was indeed seen by us in case of PARylation, which levels are modulated by the acetylation state of its the polymerase PARP1 [38]. Reversing protein deacetylation with exogenous citrate also inhibited loss of triacylglycerides, prolonged lifespan and shifted catabolism towards carbohydrate utilization. Oxaloacetate, on the other hand, was incapable of alleviating mtEcoBI effects on lifespan, demonstrating the importance of cytosolic AcCoA production for recovery. Previous attempts to replenish cytosolic AcCoA levels decreased due to starvation have been effective in correcting protein acetylation defect, but do not restore normal ATP synthesis [36]. Therefore, this metabolic inflexibility induced by translocational activity of mtEcoBI is specifically linked to protein deacetylation caused by depletion of cytosolic AcCoA and is not a passive consequence of a general deprivation of nutrients by some other mechanism.

Flies expressing the fully functional mtEcoBI with DNA cleavage activity exibited a separate mechanism that significantly augmented the inhibition of carbohydrate catabolism. It caused dephosphorylation of, and therefore inactivation of Akt kinase, which is the central regulator of glucose internalization across the plasma membrane, resulting in hyperglycemia. Due to this, the process of insulin signaling facilitating the movement of sugars into cells was quantitatively more repressed in the func than the endo- strain, adding to the metabolic inflexibility caused by a drop in cytosolic AcCoA. This led to a more rapid depletion of fat reserves and rendered the catabolism of func strain insensitive to rescue by citrate on day 6. Partial alleviation by metformin in the func but not in the endo- strain adds further proof of the dual mechanism of mtEcoBI action, which nevertheless leads to a broadly similar effect on catabolic fuel switching.

The limited effect of mtEcoBI on mtDNA in adults, accompanied by profound metabolic reprogramming aligns with findings in other mtDNA instability models [18, 20, 39]. Low-level presence of damaged mtDNA molecules doens’t necessarily mean inefficient action by mtEcoBI enzyme, as turnover of damaged DNA molecules can be very efficient in adult animals [40, 41]. Nevertheless, OXPHOS was not dysfunctional, possibly because *Drosophila* are able to endure substantial mtDNA damage without effects on respiration [42]. Therefore metabolic changes must arise via a distinct stress-response pathway.

The mtDNA is maintained *in vivo* within protein-DNA structures termed nucleoids. There is ongoing debate regarding nucleoid structure and composition [43], with a core domain generally believed to be made up of factors in direct contact with mtDNA that function in packaging, synthesis and transcription [11]. There is also a variable set of functionally diverse peripheral proteins, many having roles in metabolic pathways and some possibly in direct contact with mtDNA [44, 45]. This provides one speculative connection between mtDNA stability and metabolism, as supported by earlier findings in yeast [46, 47]. Functional loss of mtDNA maintenance proteins has been shown previously to produce metabolic defects and altered protein modifications, although the molecular mechanisms are unknown. For example, mtDNA depletion in HEK293 cells caused by loss of POLG leads to collapse of the TCA cycle and increased serine synthesis [22, 48, 49], and a mouse model of mitochondrial myopathy caused by a defective mtDNA helicase leads to major metabolic disturbances including elevated serine synthesis and disturbed one-carbon metabolism [20]. Although OXPHOS defect is a feature of these models, it is unclear if it plays an instrumental role in these metabolic abnormalities. Indeed, some of the phenotypes were reversed only by reconstitution of the TCA cycle, not by facilitating OXPHOS [48]. Interestingly, we also observed an increase in serine, together with other metabolite imbalances suggestive of elevated gluconeogenesis. As glycolysis and gluconeogenesis are reciprocally regulated, a switch from carbohydrate breakdown to storage could entail a shift towards lipid oxidation, as seen also in a mouse model [50]. Although a common finding in mitochondrial dysfunction models, the reason for serine accumulation remains unclear, and is not obligatorily linked to defective OXPHOS [23]. Our results suggest that increased serine biosynthesis may be a response to specific types of mtDNA damage or structural anomalies, underlying observed metabolic changes.

The potential of mitochondria to regulate cellular homeostasis beyond OXPHOS-related mechanisms in increasingly recognized, as several metabolites are important cofactors and regulators of a wide array of cellular functions [7]. However, direct evidence for control over metabolic flexibility has been absent so far. The apparent similarities between the effects of mtEcoBI expression in *Drosophila* adults, and various different metabolic disorders suggests that interference with mtDNA should be considered as a possible etiologic factor in diseases such as diabetes, obesity and non-alcoholic fatty liver disease (NAFLD). Such a link may have been previously overlooked due to the general assumption that mtDNA instability must be linked to OXPHOS dysfunction, to which other patological consequences are secondary. Although the underlying mechanisms that link metabolic disturbances to mtDNA remain to be elucidated, the likely relevance to disease should spur further analysis using mtEcoBI and other tools. Moreover, mtEcoBI represents a unique tool to dissect mtDNA maintenance in general and the signaling machinery that connects it with metabolism, in particular.

## Materials and methods

### Statistical methods

All measures are means of three to nine biological replicates. Error bars represent standard deviations and *p* values were calculated using two-way Student’s t-test, using Bonferroni correction where appropriate. Lifespans were analyzed using OASIS 2 online tool [51]. Descriptive analyses were performed using Kaplan-Meier estimators while Mantel-Cox tests with Bonferroni corrections were used for the pairwise comparisons of the survival functions.

### Cloning of constructs and *Drosophila* transformations

An expression cassette was amplified with Pfu PCR from pUASP plasmid [52] with oligonucleotides (5´ to 3´) UASP5Sph (ATCCAGCATGCAATTGGCCGCTCTAGCCCCC) and UASP3Nhe (ATCGGCTAGCGAGTACGCAAAGCTTGG) and inserted into SphI/SpeI-digested Green H-Pelican GFP *Drosophila* transformation vector [53]. The resulting plasmid carried a soma- and germline-competent GAL4-responsive expression cassette between *gypsy* transcriptional insulators. All three EcoBI genes were amplified by Pfu-driven PCR from *Escherichia coli* B (CGCS #2507) genomic DNA with respective chimeric primer pairs: HsdR5Eco31I (ATCGGTCTCTAATGTTATGGGCCTTAAATATTTGG) and HsdR3Xba (ATTCTAGATCAGGCCAGCTCGTCCCA); HsdS5Eco31I (ATCGGTCTCTAATGAGTTTCAACTCAACATCAAAA) and HsdS3Xba (ATTCTAGATCAGAACTTTTTACGCGA); and HsdM5Eco31I (ATCGGTCTCTAATGAACAATAACGATCTGGTCGCG) and HsdM3Xba (ATTCTAGATCATTCCTTCACCCCACC). Amplified products were digested with Eco31I and XbaI and inserted by three-fragment cloning into Not/XbaI-digested pBluescript SK^+^ along with the Eco31I/NotI-digested fragment encoding the 40 amino acid-long MTS from citrate synthase, which was amplified from *Drosophila* genomic DNA using primers CitSynCor5Not (ATGCGGCCGCATGTTCGTACGCCGTTTCGGA) and CitSynCor3Eco31 (ATCGGTCTCTCATTCGGAATGAACTTGGAGT). From the resulting plasmids, the *HsdS* and *HsdM* genes N-terminally fused to the MTS were subcloned into the customized transformation vector via NotI/XbaI digestion. The cloned *HsdR* gene was then subjected to oligonucleotide-targeted mutagenesis using the Kunkel method [54] to introduce mutations D298E and K477R. The wild-type and mutated forms of *HsdR* were inserted into the pUASTattB vector [55] via NotI/XbaI cloning. All constructs were verified by sequencing prior to use in transgenesis. Plasmid injections to create the transgenic lines were performed by Rainbow Transgenic Flies, Inc. Plasmids carrying *UAS-mtHsdM* and *UAS-mtHsdS* were inserted randomly into the *w*^*1118*^ genome via P element-mediated transgenesis, whilst *UAS-mtHsdR* was inserted into a variant *w*^*1118*^ strain bearing an attP docking site in the third chromosome (Bloomington stock number #9744). From random P-element insertions, strains were chosen that carried the *UAS-mtHsdM* and *UAS-mtHsdS* genes both on the second chromosome, and their expression was verified by qRT-PCR.

For experiments to determine the subcellular localization of the gene product in cultured S2 cells, the *HsdS*, *HsdM* and *HsdR* genes fused to the MTS from citrate synthase were amplified from pBluescript SK+ carrying the relevant construct, using M13 Reverse and HsdSXhonostop (TACTCGAGGAACTTTTTACGCGAGGCTTT), HsdMXhonostop (TACTCGAGTTCCTTCACCCCACCAAACGC) and HsdRXhonostop (TACTCGAGGGCCAGCTCGTCCCAAATGTA) primers and inserted into NotI/XhoI-digested pMT/V5-A vector DNA to create in-frame C-terminal fusions to the V5 epitope.

### *Drosophila* stocks and culture

The *Drosophila* strains carrying UAS-*mtHsdS*, *UAS-mtHsdM* and the different versions of the *UAS-mtHsdR* gene were otherwise isogenic to *w*^*1118*^. The UAS-*mtHsdS* and *UAS-mtHsdM* genes were brought together on a single second chromosome by recombination, verified by qRT-PCR after homogenizing through crossings with a CyO balancer strain. Three separate strains were then created, each carrying, in addition to *UAS-mtHsdM* and *UAS-mtHsdS*, a different *UAS-mtHsdR* isoform: wild-type (designated func), D298E (designated endo-) and K477R (designated endo-endo-/trans-) on chromosome 3 and expression of each HsdR isoform was confirmed by qRT-PCR. The presence of the three mtEcoBI binding sites was confirmed by Sanger sequencing mtDNA of all three strains using primers Dm3400 (GAATCGGCCATCAATGATATTGAAGT), Dm2350 (GTTGGAATAGATGTAGATACTCGAGCT) and Dm10800 (GTATTTACTTACATGTAGGACGAGGAA). These were then combined with different drivers. The inducible muscle- and neuronal-specific GeneSwitch driver lines (*mhcGS* and *elavGS*, respectively) were obtained from Bloomington Stock Centre (stock numbers #43641 and #43642 respectively). Other drivers used were: *daughterless GAL4* (*daGAL4*), carrying the P(GAL4-da.G32)UH1 insertion, inducible tubulin GeneSwitch (*tubGS*; kind gift from Dr. Scott Pletcher, University of Michigan). The SOD2-overexpressing line was obtained from Bloomington stock centre (#24494), whilst a mitochondrially targeted catalase-expressing strain [56] was a kind gift from Dr. Rajindar S. Sohal (USC). The ROS sensor line based on the Orp1-GFP gene fusion [57] was a kind gift from Dr. Tobias P. Dick (University of Göttingen). The hemocyte marker line expressing plasmatocyte-specific GFP and lamellocyte-specific mCherry markers [58] was obtained from Dr. Ines Anderl and Dr. Dan Hultmark (University of Umeå). Specific effects of SOD2 and mCat on larval lifespan were confirmed by using GFP co-expressing control strains (*UAS-GFP/UAS-HsdM*.*UAS-HsdS; UAS-HsdR/+*, *UAS-GFP/UAS-HsdM*.*UAS-HsdS; UAS-HsdR* D298E*/+*, *UAS-GFP/UAS-HsdM*.*UAS-HsdS; UAS-HsdR* K477R*/+*).

All *Drosophila* strains were reared and maintained at 25°C on a 12h:12h light:dark cycle on standard medium as previously described [59]. When required, different compounds were added to the medium after cooling below 65°C at the following final concentrations: mifepristone (MP) (Sigma M8046 200 μM), metformin (Acros Organics, Thermo Fisher Scientific 5 mM and 20 mM), sodium citrate (AppliChem 131655 20 mM), oxaloacetate (Alfa Aesar A12739 20 mM), L-DOPA and octopamine (Alfa Aesar A11311 3 mg/ml both).

### *Drosophila* feeding experiments

For each experiment, 150 flies were distibuted to six separate vials (25 flies per vial) with standard food and allowed to recover overnight from CO_2_ exposure. On the next day, flies from three vials were transferred without gas to standard food and from the remaining three vials to food supplemented with 1% Blue FCF dye (Acros Organics A0373695, ThermoFisher Scientific). After 2 h, 20 flies were collected and lysed by grinding in a mortar and pestle in 800 μl PBS. Debris was pelleted at 10,000 *g*_*max*_ for 10 min at 4°C and 400 μl of each supernatant were transferred to 2 wells (200 μl each) of 96-well plates. Absorbance was measured at 650 nm and values from lysates of flies kept on food without Blue FCF were used for background subtraction.

### Climbing experiment

30 males were transferred without CO_2_ anesthesia to a 300 mm long glass cylinder of 25 mm diameter. The distance travelled after collecting flies to the bottom by tapping the container was measured after 4 seconds, from video recordings viewed in slow-motion.

### Wet weight measurements

*Drosophila* parental strains (approximately 300 females and 150 males) were mated in bottles for 24 h and then transferred to mating chambers (Genesee #59–101) with standard food plates supplemented with yeast paste (yeast extract mixed with water) to facilitate egg laying. Developing embryos were collected after 2 h and 30–60 larvae depending on the developmental stage were isolated and weighed. Data from each timepoint were obtained in three biological replicates.

### Agarose gel electrophoresis and Southern blotting

Batches of several hundred flies were homogenized in a Dounce homogenizer as previously [60] and nucleic acid was extracted from mitochondria (omitting the sucrose-gradient centrifugation). For 1D DNA electrophoresis, 1 μg of mtDNA was loaded per lane in 0.5% agarose gels run in TBE without ethidium bromide for 20 h at 0.7 V/cm. For topoisomerase treatment 1 μg of mtDNA was incubated with Topo I (New England Biolabs, M0301S) under conditions recommended by the manufacturer. 1D gels were stained with ethidium bromide and documented, prior to blotting. For 2DNAGE, 10 μg aliquots of mtDNA digested with ClaI (4 U/μg for 4 h at 37°C) were eletrophoresed as previously [60]. Southern blotting and hybridization were as described previously, using probe 12 [60]. Different topological forms were quantified using ImageLab software from BioRad.

### qPCR and qRT-PCR

For qPCR measurement of mtDNA copy number total DNA was isolated from 20 flies using QIAGEN DNeasy Blood and Tissue kit, following the supplementary protocol for insect samples. For cDNA synthesis, 40 flies were homogenized on ice in 1 ml Trizol. After 5 min incubation at room temperature, 200 μl of chloroform was added. After mixing and further incubation at room temperature for 3 min, debris was pelleted and phases separated by centrifugation of 12,000 *g*_*max*_ for 15 min at 4°C. 600 μl of the upper phase were decanted and mixed with 500 μl of isopropanol. After incubation for 10 min at room temperature nucleic acids were pelleted by centrifugation of 12,000 *g*_*max*_ for 10 min at 4°C. Pellets were washed once with 75% ethanol and resuspended in DEPC-treated water with subsequent incubation at 55°C for 10 min. 20 μl of DNase I buffer along with 1 U of DNase I (Thermo Fisher Scientific, EN0521) were added and samples were incubated for 1 h at 37°C. After extraction once with phenol:chloroform:isoamylalcohol (25:24:1) and once with chloroform, RNA was ethanol precipitated overnight at -20°C and was recovered by centrifugation at 10,000 *g*_*max*_ for 10 min at 4°C, washed once with 70% ethanol and redissolved in 100 μl of DEPC-treated water. 2 μg were used for cDNA synthesis with random hexameric primers, using High-Capacity cDNA Reverse Transcription Kit (Applied Biosystems) according to manufacturer’s protocol. For quantitative PCR analysis of mtDNA copy number 10 ng of total DNA was used for reactions that were carried out in StepOnePlus^™^ Real-Time PCR System with Fast SYBR Green Master Mix kit (Applied Biosystems). Primer sequences can be found in S1 Table.

### Blue-native gel electrophoresis

Larvae were collected from egg-laying plates (as for wet-weight measurement) and gently Dounce-homogenized on ice in 600 μl of homogenization buffer (250 mM sucrose, 2 mM EGTA, 0.1% BSA, 5 mM Tris/HCl, pH 7.4). The supernatant from centrifugation at 200 *g*_*max*_ for 3 min at 4°C was re-centrifuged at 9,000 *g*_*max*_ for 5 min at 4°C. The crude mitochondrial pellet was resuspended in homogenization buffer without BSA and protein concentration was measured with Bradford assay. 100 μg aliquots of the lysate were centrifuged at 9,000 *g*_*max*_ for 5 min at 4°C and pellets were resuspended in 25 μl of NativePAGE sample buffer (50 mM NaCl, 10% glycerol, 0.001% Ponceau S, 50 mM BisTris/HCl pH 7.2) containing 1% digitonin (D5628 Sigma) and protease inhibitor cocktail (Roche Complete Mini #11836170001). After incubating on ice for 15 min, samples were centrifuged at 16,100 *g*_*max*_ for 30 min at 4°C. 25 μl of supernatant were mixed with with 1.5 μl G-250 sample additive (Thermo Fisher Scientific), 10 μl 4 x NativePAGE sample buffer and 3.5 μl water. 30 μg aliquots of protein were loaded onto NativePAGE BisTris gels (Invitrogen). The inner chamber of the running apparatus was filled with 1 x dark blue cathode buffer (50 mM BisTris; 50 mM Tricine; 0.02% G-250 dye) and the outer chamber with 1 x anode buffer (50 mM BisTris, 50 mM Tricine, pH 6.8). Samples were run at 70 V for approximately 1 h at 4°C until the dye front had migrated approximately 1/3 of the way through the gel, after which the cathode buffer was replaced with light blue cathode buffer (50 mM BisTris; 50 mM Tricine; 0.002% G-250 dye). Samples were run for a further 21 h at 25 V and 4°C. After the run gel was subjected to staining either for complex I or for IV activity. Before staining, gels were equilibrated in the appropriate reaction buffers without chromogenic agents for 10 min at room temperature. After equilibration, gels were incubated in reaction buffers containing chromogenic agents for variable times, as follows. For complex I staining, the reaction buffer contained 5 mM Tris/HCl (pH 7.4), 2.5 mg/ml nitrotetrazolium blue and 0.1 mg/ml NADH. For complex IV, the reaction buffer contained 50 mM sodium phosphate (pH 7.2), 0.05% 3.3′-diaminobenzidine tetrahydrochloride and 50 mM horse-heart cytochrome c (C2867 Sigma).

### Respirometry

For adults, 100 male flies were pooled into one food bottle and left to recover from CO_2_ exposure for at least 24 h. For developing *Drosophila*, 100–200 larvae were collected from egg-laying plates, as for wet-weight measurement. Animals were transferred into a chilled mortar and gently Dounce-homogenized (30 strokes) on ice in 500 μl homogenization buffer (250 mM sucrose, 2 mM EGTA, 5 mM Tris/Hcl, pH 7.4). Lysates were filtered through 200 μm nylon mesh rinsed with a further 500 μl of homogenization buffer at 4°C. 25 μl of larval lysate was transferred to the chamber of an Oroboros O2K oxygraph containing 1975 μl of respiration buffer (120 mM KCl, 1 mM EGTA, 1 mM MgCl_2_, 0.2% BSA, 5 mM KH_2_PO_4_, 3 mM Hepes/KOH, pH 7.2). Oxygen consumption was measured after sequential additions of substrates and inhibitors at the following final concentrations: proline (5 mM), pyruvate (5 mM), ADP (1 mM), rotenone (0.5 μM), glycerol-3-phosphate (20 mM), antimycin (2.5 μM), ascorbate (2 mM), *N*,*N*,*N′*,*N′*-tetramethyl-*p*-phenylenediamine (0.5 mM), potassium cyanide (1 mM). 25 μl of fly lysates were assayed using a Hansatech Oxytherm respirometer containing 475 μl respiration buffer, with oxygen consumption measured after sequential additions of substrates and inhibitors at the following final concentrations: proline (10 mM), pyruvate (10 mM), ADP (1 mM), rotenone (150 nM), glycerol-3-phosphate (10 mM), antimycin (0.1 μM), ascorbate (10 mM), *N*,*N*,*N′*,*N′*-tetramethyl-*p*-phenylenediamine (10 mM), potassium cyanide (200 μM). Substrate and inhibitor concentrations were pre-calibrated to fit the specific apparatuses used. Values were normalized to protein concentration measured by the Bradford assay. RER was calculated as a ratio of CO_2_ produced and O_2_ used by flies. For this, O_2_ consumption in individual living flies was measured by coulometric respirometry in a continuous O_2_-compensating system at constant temperature and humidity (23°C and 55% RH). Flies were placed into measuring chambers and measurements were started when the flies stopped moving and the minimum value of gas exchange was reached. CO_2_ levels were determined using a LI-700 differential CO_2_/H_2_O analyzer (LiCor, Lincoln, Nebraska, USA).

### Microscopy

To visualize hemocytes and GFP-based ROS signal, larvae were carefully washed in water using a fine paint-brush, dried on tissue paper and embedded on microscope slides in a drop of ice-cold 80% glycerol. The larvae were immobilized at -20°C for 24 h before live imaging using a Zeiss ApoTome.2 structured illumination microscope. For subcellular localization by immunocytochemistry, *Drosophila* S2 cells were transformed with pMT/V5-His A constructs using Fugene HD (Promega) according to manufacturer’s protocol. After induction with 500 μM Cu_2_SO_4_ for 48 h, cells were fixed and V5-tagged proteins and endogenous COXIV were detected as described previously [61], using mouse anti-V5 (Life Technologies, 1:1000) and rabbit anti-COXIV (Abcam, 1:300) as primary antibodies, respectively with AlexaFluor 568 goat anti-mouse IgG (H+L) and goat anti-rabbit AlexaFluor 488 IgG (H+L) (Life Technologies) as secondary antibodies (1:1000 in both cases). Samples were mounted in ProLong Gold Antifade Mountant with DAPI (ThermoFisher Scientific, P36931), according to manufacturer’s protocol.

### Western sample preparation and blotting

Batches of 30 flies were homogenized with a pestle on ice in 300 μl of western lysis buffer (PBS with 1.5% Triton X-100) supplemented with protease and phosphatase inhibitor cocktails (Roche Complete Mini #11836170001 and PhosSTOP #04906845001) following manufacturer's protocols. For accetylation analysis inhibitors of deacetylases (10 mM sodium butyrate, 20 mM nicotinamide and 20 nM trichostatin A) were added to the lysis buffer. Lysates were incubated on ice for 15 min and then centrifuged at 13,000 *g*_*max*_ for 15 min at 4°C to pellet debris. Supernatant protein concentrations were measured using the Bradford assay and 50 μg aliquots were loaded onto precast Bio-Rad Criterion 7.5% (for HsdR) or 12% acrylamide (for eIF2α and phospho eIF2α) or BioRad AnyKD gradient gels (for other proteins). Gels were run in ProSieve EX running buffer (Lonza). Proteins were transferred to Amersham Protran nitrocellulose membrane (#10600020) in ProSieve EX transfer buffer (Lonza) at 35 V for 50 min in a BioRad Criterion Transfer chamber. Membranes were incubated in blocking and antibody buffers with appropriate antibody concentrations as detailed in S2 Table. Secondary antibody was always conjugated with horseradish peroxidase. Results were visualized either on autoradiography film or with BioRad ChemiDoc XR detection system. For quantitation, samples from all three strains to be compared were run on the same gel with 4 individual biological replicates per strain. When protein amount per lane was used for normalization, membranes were stained with Ponceau S solution (0.1% Ponceau S in 5% acetic acid), rinsed briefly with water and documented using the BioRad ChemiDoc XR system. Signal was quantified and data analyzed with ImageQuant software. Protein signal linearity of Ponceau S-stained membranes between 10 and 80 μg was confirmed (S14A Fig). Western blots and corresponding Ponceau S-stained membranes used for quantifications are presented in S14B Fig. Protein sample from *E*.*coli* B-strain bacteria used in S1D Fig was prepared using B-PER Bacterial Protein Extraction Reagent (Thermo Fisher Scientific, 78248) following manufacturer’s protocol.

### Brain DHE staining

Brains were dissected from adult flies at room temperature in *Drosophila* Schneider’s cell medium (SCM) supplemented with L-glutamine, then incubated in SCM with 30 μM dihydroethidium (Thermo Fisher Scientific, D11347) on a nutator for 7 min in the dark. Following a rinse with SCM, brains were washed 3 x 5 min with SCM, then placed on a glass slide between double-sided tape strips and covered with coverglass. Vectashield mounting medium (Vector #H-1000) was infused under the coverglass and brains were immediately scanned at 10x magnification using a Zeiss LSM 780 confocal laser-scanning microscope. Maximum intensity projections were created with ImageJ software.

### Ovary dissections

Ovaries from *elavGS*>mtEcoBI flies 45 d after eclosion were dissected in PBS on a depression slide. Images were captured directly, using a Nikon SMZ745T camera at 60x total magnification.

### Lifespan and developmental time measurements

For lifespan measurements, 20 male flies were collected no later than 72 h after eclosion and placed in food vials with at least three vials per measurement. Flies were transferred to fresh vials three times a week and viability was recorded until the last fly had died. For developmental time measurements, 6 replicates of 20 females with 10 males were premated and then allowed to lay eggs for 24 h, after which the eclosion day of progeny was scored.

### Metabolite measurements

Pyruvate and lactate were measured using BioVision kits (#K709 and #K607) according to modified protocols provided by the manufacturer. For pyruvate measurements, 20 flies were homogenized in 200 μl Pyruvate Assay Buffer on ice and then centrifuged at 10,000 *g*_*max*_ for 10 min at 4°C. 15 μl of supernatant was mixed with 35 μl of Pyruvate Assay buffer in a well of 96-well microtiter plate. 50 μl of reaction mix (formulated according to manufacturer’s guidelines) was added to every well containing supernatant, incubated for 30 min at room temperature after which absorption was measured at 570 nm. For the standard curve, pyruvate concentrations between 2 and 10 nM were used. Parallel background reactions were performed by mixing supernatant with background mix, formulated according to manufacturers’ guidelines. For lactate measurements, 20 flies were homogenized in 100 μl PBS on ice and incubated at 65°C for 15 minutes. Debris was pelleted by brief centrifugation and 10 μl of lysate was assayed according to the manufacturers’ protocol similar to pyruvate measurement. For the lactate standard curve concentrations between 100 and 1000 pM were used. ATP concentration was measured using the ATP Determination Kit (ThermoFisher Scientific). 30 flies were homogenized in ATP isolation buffer (6M guanidine-HCl, 4 mM EDTA, 100 mM Tris/Cl pH 7.8) and snap-frozen in liquid nitrogen, followed by boiling for 5 min. Debris was pelleted by centrifugation at 10,000 g_max_ for 10 min at 4°C. 5 μl of a 12.5-fold diluted supernatant was added to 100 ul of ATP Reaction Mix (formulated according to manufacturers’ recommendations) and values were recorded using a Tecan luminometer with Greiner polypropylene plates (#655207). Total NAD and the NAD^+^/NADH ratio were determined with Sigma kit MAK037. 20 flies were homogenized in 400 μl of extraction buffer and centrifuged at 10,000 g_max_ for 5 min at 4°C. Supernatants were filtered through 10 kDa Spin Columns (Abcam ab93349) with centrifugation at 18,000 *g*_*max*_ for 20 min at 4°C and divided into two equal portions. One portion was heated at 60°C for 30 min. 10 μl of the heat-treated and untreated samples were distributed to wells of a microtitre plate, Volumes were brought up to 50 μl with reaction buffer and mixed with 100 μl of reaction mix, consisting of NAD cycling buffer and cycling enzyme, formulated according to manufacturer’s guidelines. Reactions were incubated at room temperature for 5 min, after which 10 μl of NADH developer was added to each well and cycling measurement was started at OD 450 nm.

AcCoA, propanoyl-CoA, fumarate, malate, citrate, isocitrate, PEP, aspartate, glutamate, ketogenic amino acids and L-DOPA were measured by capillary electrophoresis-mass spectrometry method by Human Metabolome Technologies (Japan). Flies were collected without CO_2_ gas, frozen and sent to HMT on dry ice. There samples were mixed with 1,500 μl of 50% acetonitrile in water (v/v) containing internal standards (20 μM for cation and 5 μM for anion measurement) and homogenized. Supernatant was filtered through 5-kDa cut-off filter, concentrated by centrifugation and resuspended in 50 μl of water before measurement using fused silica capillary 50 μm x 80 cm. For cationic metabolites conditions were as follows: pressure injection: 50 mbar for 10 sec, voltage: 27 kV, ionization: positive, capillary voltage: 4,000 V, scan range: *m/z* 50–1000. For anionic metabolites conditions were as follows: pressure injection: 50 mbar for 25 sec, voltage: 30 kV, ionization: negative, capillary voltage: 3,500 V, scan range: *m/z* 50–1000. Peaks were extracted with MasterHands sofware ver. 2.17.1.11 and putative metabolites were assigned from HMT’s standard library with tolerance of ± 0.5 min for migraton time and ±10 ppm for *m/z*.

### Hemolymph glucose measurements

Thoraxes of 100 flies were punctured by a 20-gauge needle and flies were transferred to 0.2 ml Eppendorf tubes with a punctured bottom. Tubes were placed within 0.5 ml Eppendorf tubes and centrifuged at 1,500 *g*_*max*_ for 5 min at 4°C. 2 μl of supernatant was collected from the bottom of each tube and mixed with 8 μl of TBS (pH 6.6) with subsequent incubation at 70°C for 5 min. 5 mU of trehalase (T8778 Sigma) was added, followed by overmight incubation at 37°C and the addition of 100 μl Glucose Assay Reagent (G3293 Sigma). After incubation at 37°C for 30 min absorbance was measured at 340 nm.

### Carbohydrate and triglyceride measurements

Carbohydrate and triglyceride measurements were performed as described previously [62].

**Carbohydrate measurements.** 10 flies were homogenized in 400 μl of PBS and incubated for 5 min at 70°C. 40 μl of lysate was transferred to four separate Eppendorf tubes with additions of 1U of amyloglycosidase from *Aspergillus niger* (Sigma, total glucose measurement), 2 x PBS (free glucose and background measurement) and 5 mU of porcine kidney trehalase (T8778 Sigma, trehalose measurement). All reactions were incubated for 2 h at 37°C, after which they were briefly centrifuged and 30 μl of supernatant was transferred to 96-well microtiter plates. 100 μl of Glucose Assay Reagent (Sigma G3293) was added to all reactions except for one PBS-treated lysate that was mixed with 100 μl of PBS to measure background. Reactions were incubated at 37°C for 30 min, after which absorption was measured at 340 nm. Free glucose, glycogen and trehalose were calculated by subtracting relevant backgrounds from measured values. A glucose standard curve was generated using 1 to 20 μg of glucose (per well).

**Triglyceride measurements.** 20 flies were homogenized in 800 ul of PBS with 0.1% Tween 20 and incubated for 5 min at 70°C. 20 μl of each lysate was transferred to three separate Eppendorf tubes with additions of 20 μl of Triglyceride Reagent (Sigma T2449, total glycerol measurement) and 2 x 20 μl of PBS (free glycerol and background measurement). All reactions were incubated for 30 min at 37°C, after which they were briefly centrifuged and 30 μl of supernatant was transferred to 96-well microtiter plates. 100 ul of Free Glycerol Reagent (Sigma F6428) was added to all reactions except for one PBS-treated lysate that was mixed with 100 μl of PBS to measure background. Reactions were incubated at 37°C for 5 min, after which absorption was measured at 540 nm. Triglycerides were calculated by subtracting free glycerol from total glycerol measurement. A glycerol standard curve was calculated by using 0.5 μg to 3 μg of glycerol (per well).

### Protein carbonylation measurements

Oxidative stress-derived protein damage markers (for protein glycoxidation (N^ε^-(carboxyethyl)-lysine [CEL]), for lipoxidation (N^ε^-malondialdehyde-lysine [MDAL], and for mixed glyco-/lipoxidation (N^ε^-(carboxymethyl)-lysine [CML] and N^ε^-(carboxymethyl)-cysteine [CMC])) were determined as trifluoroacetic acid methyl ester (TFAME) derivatives in acid hydrolyzed, delipidated and reduced protein samples by GC/MS using a HP6890 Series II gas chromatograph (Agilent, Barcelona, Spain) with a MSD5973A Series detector and a 7683 Series automatic injector, a HP-5MS column (30-m x 0.25-mm x 0.25-μm). The injection port was maintained at 275°C; the temperature program was 5 min at 110°C, then 2°C/min to 150°C, then 5°C/min to 240°C, then 25°C/min to 300°C, and finally held at 300°C for 5 min. Quantitation was performed by internal and external standardization using standard curves constructed from mixtures of deuterated and non-deuterated standards. Analyses were carried out by selected ion-monitoring GC/MS (SIM-GC/MS). The ions used were: lysine and [^2^H_8_]lysine, m/z 180 and 187, respectively; CEL and [^2^H_4_]CEL, m/z 379 and 383, respectively; CML and [^2^H_4_]CML, m/z 392 and 396, CMC and [^13^C_2_]CMC, m/z 271 and 273, respectively; MDAL and [^2^H_8_]MDAL, m/z 474 and 482, respectively. The amounts of product were expressed as μmoles of CEL, CML, MDAL or CMC per mol of lysine.

### Enzyme activity measurements

All enzyme activity measurements were carried out using relevant BioVision assay kits (#K776 for phosphofructokinase, #K789 for hexokinase, #K709 for pyruvate kinase) and ApexBio assay kit for succinate dehydrogenase (#K2210).

#### PFK measurements

10 flies were homogenized in 200 μl Phosphofructokinase Assay buffer on ice and centrifuged at 10,000 g_max_ for 5 min at 4°C. Supernatants were diluted 1:10 with Phosphofructokinase Assay buffer and 1 μl was mixed with reaction mix formulated according to manufacturers’ guidelines. Parallel background reactions were performed by mixing supernatant with background mix according to manufacturer’s guidelines. Absorption was measured immediately in kinetic mode for 60 min at 2 min intervals at 450 nm. An NADH standard curve was generated using NADH concentrations from 2–10 nmol.

#### Hexokinase measurements

10 flies were homogenized in 400 μl of Hexokinase Assay buffer on ice, incubated on ice for 10 minutes and centrifuged with 10,000 x g_max_ for 5 min at 4°C. 1 μl of supernatant was mixed with reaction mix formulated according to manufacturers’ guidelines. Parallel background reactions were performed by mixing supernatant with background mix formulated according to manufacturers’ guidelines. Absorption was measured immediately with kinetic mode for 60 min with 2 minute intervals at 450 nm. An NADH standard curve was generated using NADH concentrations from from 2.5 to 12.5.

#### Pyruvate kinase measurements

10 flies were homogenized in 200 μl of Pyruvate Kinase Assay buffer on ice and centrifuged with 10,000 x g_max_ for 5 min at 4°C. 1 μl of supernatant was mixed with reaction mix formulated according to manufacturers’ guidelines. Parallel background reactions were performed by mixing supernatant with background mix formulated according to manufacturers’ guidelines. Absorption was measured immediately with kinetic mode for 60 min with 2 min intervals at 570 nm. A pyruvate standard curve was generated using pyruvate concentrations from from 200 nmol to 1000 nmol.

#### Pyruvate dehydrogenase measurements

10 flies were homogenized in 200 μl of Pyruvate Dehydrogenase Assay buffer on ice, incubated on ice for 10 min and centrifuged with 10,000 x g_max_ for 5 min at 4°C. 10 μl of supernatant was mixed with reaction mix formulated accordign to manufacturers’ guidelines. Parallel background reactions were performed by mixing supernatant with background mix formulated according to manufacturers’ guidelines. Absorption was measured immediately with kinetic mode for 60 min with 2 min intervals at 450 nm. An NADH standard curve was generated using NADH concentrations from from 2 to 10 nmol.

#### Succinate dehydrogenase measurements

10 flies were homogenized in 100 ul of Assay buffer, incubated on ice for 10 min and then centrifuged for 5 min at 10,000 x g_max_. 40 ul of supernatant was mixed with reaction mix formulated according to manufacturers’ guidelines. Absorption was measured immediately with kinetic mode for 30 min with 1 min intervals at 600 nm. A DCIP standard curve was generated using DCIP concentrations according to manufacturers’ protocol.

## Supporting information

S1 Table Oligonucleotides used in qRT-PCR. (PDF)

S2 Table Antibody dilutions and incubation conditions. (PDF)

S1 Text Supporting Information references. (PDF)

S1 Dataset Numerical data. (XLSX)

S1 Fig Expression and localisation of early-onset expression of mtEcoBI. **(A)** Steady-state mRNA levels of HsdM, HsdS and HsdR subunits after induction with 200 μM mifepristone for 6 days either in strain carrying the methyltransferase-capable form of mtEcoBI consisting only of HsdM and HsdS subunits (*UAS-mtHsdM*.*UAS-mtHsdS*/+;*tubGS/+*) or in strains carrying the full enzyme isoforms (*UAS-mtHsdM*.*UAS-mtHsdS*/+;*UAS-mtHsdR*/*tubGS*, *UASmtHsdM*.*UAS-mtHsdS*/+;*UAS-mtHsdR* D298E/*tubGS*, *UAS-mtHsdM*.*UAS-mtHsdS*/+;*UAS-mtHsdR* K477R/*tubGS*). *p*<0.0001 (****), n = 5. **(B)** Subcellular localisation of HsdS fused to citrate synthase MTS and to V5-epitope in transiently expressing S2 cells. Cells were stained for DAPI and labeled with antibodies against COXIVand V5-epitope, followed by incubation with secondary antibodies conjugated with Alexa 568 (green) and Alexa 488 (red) respectively. Scale bar is 5 mm. **(C)** Subcellular fractionation of *Drosophila* tissue from *UAS-mtHsdM*.*UAS-mtHsdS*/+;*UAS-mtHsdR*/*tubGS* strain after 6 days of incubation either on regular or 200 μM mifepristone-containing food using HsdR, Akt (cytosolic marker), NDUFS3 (mitochondrial marker) and histone 3 (nuclear marker) antibodies, u—uninduced, i—induced. **(D)** Western of *Drosophila* strains expressing different isoforms of HsdR subunit with antibodies against HsdR, *E*.*coli* B-strain served as a control. Ponceau S-stained membrane was used as a loading control.
(PDF)

S2 Fig Schematic map of *D*. *melanogaster* mtDNA and effects of early-onset expression of mtEcoBI. **(A)** Schematic map of *Drosophila* mtDNA showing major mtEcoBI cleavage sites (red arrows), positions of mTTF/mTERF5 binding sites (mTTF bs1 and bs2), positions of genes used for transcript measurements (in blue), non-coding region (NCR) and origin of replication (arrow within NCR). **(B)** Sequences of mtDNA regions containing mtEcoBI binding sites TGA-(N)_8_-TGCT (blue arrows) in strain *UAS-mtHsdM*.*UAS-mtHsdS*/+;*UAS-mtHsdR*/*daGAL4* 3 days after egg laying. Numbers refer to position of nucleotides in *D*. *melanogaster* mtDNA in NCBI nucleotide databank entry NC_001709. **(C)** 1D gel electrophoresis of uncut mtDNA samples from larvae of endo/trans- (*UAS-mtHsdM*.*UAS-mtHsdS*/+;*UAS-mtHsdR* K477R/*daGAL4*), endo- (*UASmtHsdM*.*UAS-mtHsdS*/+;*UAS-mtHsdR* D298E/*daGAL4*) and func (*UAS-mtHsdM*.*UASmtHsdS*/+;*UAS-mtHsdR*/*daGAL4*) strains used for quantifications of covalently closed (cc) forms of different linking number in Fig 1D. **(D)** State III respiration of mitochondria isolated from *UASmtHsdM*.*UAS-mtHsdS*/+;*UAS-mtHsdR*/*daGAL4*, *UAS-mtHsdM*.*UAS-mtHsdS*/+;*UAS-mtHsdR* D298E/*daGAL4*, *UAS-mtHsdM*.*UAS-mtHsdS*/+;*UAS-mtHsdR* K477R/*daGAL4* strain larvae day 2 AEL, *p*<0.01 (**), n = 3. **(E)** Wet weight of *UAS-mtHsdM*.*UAS-mtHsdS*/*UAS-SOD2*;*UAS-mtHsdR*/*daGAL4*, *UASmtHsdM*.*UAS-mtHsdS*/*UAS-SOD2*;*UAS-mtHsdR* D298E/*daGAL4*, *UAS-mtHsdM*.*UASmtHsdS*/ *UAS-SOD2*;*UAS-mtHsdR* K477R/*daGAL4* and *UAS-mtHsdM*.*UAS-mtHsdS*/*UASmCat*; *UAS-mtHsdR*/*daGAL4*, *UAS-mtHsdM*.*UAS-mtHsdS*/*UAS-mCat*;*UAS-mtHsdR* D298E/*daGAL4*, *UAS-mtHsdM*.*UAS-mtHsdS*/*UAS-mCat*;*UAS-mtHsdR* K477R/*daGAL4* and *UAS-mtHsdM*.*UASmtHsdS*/*UAS-GFP*;*UAS-mtHsdR*/*daGAL4*, *UAS-mtHsdM*.*UAS-mtHsdS*/*UAS-GFP*;*UAS-mtHsdR* D298E/*daGAL4*, *UAS-mtHsdM*.*UAS-mtHsdS*/*UAS-GFP*;*UAS-mtHsdR* K477R/*daGAL4* larvae. Days mark time after egg laying, n = 3.
(PDF)

S3 Fig ROS-induced overproduction of lamellocytes. **(A)** Microscopy of *mo-mCherry eater-MSNF9 GFP*/*FM7a*;*UAS-mtHsdM UASmtHsdS*/+;*UAS-mtHsdR* D298E/*daGAL4* (endo-) and *MSNF9mo-mCherry eater-GFP*/*FM7a*;*UASmtHsdM*.*UAS-mtHsdS*/+;*UAS-mtHsdR* K477R/*daGAL4* (endo/trans-) L3 larvae (5 days after egg laying) showing green plasmatocytes and red lamellocyte signal. White arrows point to the red signalin larval muscle that is caused by labeling artefact of the given reporter system [1]. Scale bar is 0,5 mm. **(B)** *UAS-mtHsdM*.*UAS-mtHsdS*/+;*UAS-mtHsdR* D298E/*daGAL4* (endo-) larvae of L3 stage (5 days after egg laying) showing melanotic nodules reared on regular food (left) and on food supplemented with 1,5 mM N-acetyl cysteine. „SOD2 overexpression” refers to *UAS-mtHsdM*.*UASmtHsdS*/*UAS-SOD2*;*UAS-mtHsdR* D298E/*daGAL4* (endo-) larvae grown on regular food. Scale bar is 1 mm.
(PDF)

S4 Fig Phenotype and mtEcoBI binding sequences of flies with adult-onset expression of mtEcoBI variants and subunits. **(A)** Lifespans of *tubGS*>mtEcoBI endo/trans- (*UAS-mtHsdM*.*UAS-mtHsdS*/+;*UASmtHsdR* K477R/*tubGS*) and *w1118* strains on food with and without 200 μM mifepristone. **(B)** Lifespans of strains expressing different combinations of mtEcoBI subunits from *tubGS* driver (M+S: *UAS-mtHsdM*.*UAS-mtHsdS*/+;*tubGS*/+, R (K477R): *UAS-mtHsdR*/*tubGS*, R (D298E): *UAS-mtHsdR* D298E/*tubGS*, R (wt): *UAS-mtHsdR* K477R/*tubGS*). **(C)** Developmental time comparison between *w*^*1118*^ and endo/trans-, ns–not significant, n = 5. **(D)** Climbing activities of flies from *tubGS*>mtEcoBI strains at days 6 and 7 after induction with 200 μM mifepristone, *p*<0.01 (**), *p*<0.0001 (****), n = 18–32. **(E)** Sequences of mtDNA regions containing mtEcoBI binding sites TGA-(N) -TGCT (blue arrows) in *tubGS*>mtEcoBI func strain (*UAS-mtHsdM*.*UAS-mtHsdS*/+;*UAS-mtHsdR*/*tubGS*) with or without induction with 200 μM MP for 10 days. Numbers refer to position of nucleotides in *D*. *melanogaster* mtDNA in NCBI nucleotide databank entry NC_001709.
(PDF)

S5 Fig Two-dimensional analysis of mtDNA replication intermediates from *tubGS*>mtEcoBI strains. **(A)** 2DNAGE of mtDNA Cla fragment (nt 7874–12951 in NC_001709) from *tubGS*>mtEcoBI endo/trans- ((*UAS-mtHsdM*.*UAS-mtHsdS*/+;*UAS-mtHsdR* K477R/*tubGS*), endo- (*UASmtHsdM*.*UAS-mtHsdS*/+;*UAS-mtHsdR* D298E/*tubGS*) and func (*UAS-mtHsdM*.*UAS-mtHsdS*/+;*UASmtHsdR*/*tubGS*) strains kept 10 days on food with or without 200 μM MP (induced/uninduced). **(B)** Drawing detailing major replication and recombination intermediates separated on 2DNAGE on panel A.
(PDF)

S6 Fig Ponceau S-stained membranes of porin and NDUFS3 westerns. Total protein amount visualized with Ponceau S—staining from *tubGS*>mtEcoBI. endo/trans- (*UAS-mtHsdM UAS-mtHsdS*/+;*UAS-mtHsdR* K477R/*tubGS*), endo- (*UAS-mtHsdM*.*UAS-mtHsdS*/+;*UAS-mtHsdR* D298E/*tubGS*) and func (*UAS-mtHsdM UAS-mtHsdS*/+;*UASmtHsdR*/*tubGS*) strains. AI: after induction on 200 μM MP.
(PDF)

S7 Fig ROS levels in dissected brains and effect of SOD2/mCat overexpression on lifespans of *tubGS*>mtEcoBI strains. **(A)** Maximum projections of dihydroethidium (DHE)-stained brains from indicated strains after 6 days of induction with 200 μM MP. Scale bar 100 μm. **(B)** Lifespans of indicated strains with co-overexpression of either SOD2 (*UAS-mtHsdM*.*UAS-mtHsdS*/*UAS-SOD2*;*UASmtHsdR*/*tubGS* and *UAS-mtHsdM*.*UAS-mtHsdS*/*UAS-SOD2*;*UAS-mtHsdR* D298E/*tubGS*) or mCat (*UAS-mtHsdM*.*UAS-mtHsdS*/*UA*S-mCat;*UAS-mtHsdR/tubGS* and *UAS-mtHsdM*.*UASmtHsdS/UAS-*mCat;*UAS-mtHsdR* D298E/*tubGS*).
(PDF)

S8 Fig Expression of mtUPR and ISR markers in *tubGS*>mtEcoBI strains. (A) Steady-state mRNA levels of several mtUPR markers from *tubGS*>mtEcoBI endo/trans- (*UAS-mtHsdM.UAS-mtHsdS*/+;*UAS-mtHsdR* K477R/*tubGS*), endo- (*UASmtHsdM.UAS-mtHsdS*/+;*UAS-mtHsdR* D298E/*tubGS*) and func (*UAS-mtHsdM.UASmtHsdS*/+;*UAS-mtHsdR/tubGS*) strains after 10 days of induction with 200 μM MP, n = 3. (B) Ratio of total eIF2a to phosphorylated eIF2a from *tubGS*>mtEcoBI endo/trans- ((*UAS-mtHsdM.UASmtHsdS*/+;*UAS-mtHsdR* K477R/*tubGS*), endo- (*UAS-mtHsdM.UAS-mtHsdS*/+;*UAS-mtHsdR* D298E/*tubGS*) and func (*UAS-mtHsdM.UAS-mtHsdS*/+;*UAS-mtHsdR/tubGS*) strains after 10 days of induction with 200 μM MP, ns—not significant, n = 4. Westerns used for quantifications are shown on the right.
(PDF)

S9 Fig Levels of central carbon metabolites in *tubGS*>mtEcoBI strains. Levels of propanoyl-CoA and TCA intermediates from *tubGS*>mtEcoBI endo/trans-(*UAS-mtHsdM*.*UAS-mtHsdS*/+;*UAS-mtHsdR* K477R/*tubGS*), endo- (*UAS-mtHsdM*.*UASmtHsdS*/+;*UAS-mtHsdR* D298E/*tubGS*) and func (*UAS-mtHsdM*.*UAS-mtHsdS*/+;*UASmtHsdR*/*tubGS*) strains after 6 days of induction with 200 μM MP., *p*<0.05(*), *p*<0.01 (**), *p*<0.001 (***), *p*<0.0001 (****), n = 3 or 5.
(PDF)

S10 Fig Lipid and carbohydrate stores, Akt westerns and effect of metformin to lifespan in *tubGS*>mtEcoBI strains. **(A)** Comparison on TAG between age-matched *tubGS*>MTase ((*UAS-mtHsdM*.*UASmtHsdS*/+;*tubGS*/+) strains and between *w*^*1118*^ and *tubGS*>mtEcoBI endo/trans- (*mtHsdS*/+;*UAS-mtHsdR* K477R/*tubGS*) strains after 10 days either on regular or MP-supplemented (200 μM) food, ns–not significant, n = 5. **(B)** Levels of carbohydrates from *tubGS*>mtEcoBI endo/trans- (*UAS-mtHsdM*.*UAS-mtHsdS*/+;*UAS-mtHsdR* K477R/*tubGS*), endo- (*UAS-mtHsdM*.*UASmtHsdS*/+;*UAS-mtHsdR* D298E/*tubGS*) and func (*UAS-mtHsdM*.*UAS-mtHsdS*/+;*UASmtHsdR*/*tubGS*) strains (normalized to protein content) after 6 days of induction with 200 μM MP, *p*<0.05 (*), n = 5. **(C)** Westerns of *tubGS*>mtEcoBI endo/trans- ((*UAS-mtHsdM*.*UAS-mtHsdS*/+;*UAS-mtHsdR* K477R/*tubGS*), endo- (*UAS-mtHsdM*.*UAS-mtHsdS*/+;*UAS-mtHsdR* D298E/*tubGS*) and func (*UAS-mtHsdM*.*UAS-mtHsdS*/+;*UAS-mtHsdR*/*tubGS*) flies with pan-Akt and phospho-Akt antibodies after 10 days of induction with 200 μM MP used in quantifications shown in Fig 3H. **(D)** Lifespans of *tubGS*>mtEcoBI endo- (*UAS-mtHsdM*.*UAS-mtHsdS*/+;*UAS-mtHsdR* D298E/*tubGS*) and endo/trans- (*UAS-mtHsdM*.*UAS-mtHsdS*/+;*UAS-mtHsdR* K477R/*tubGS*) strains with *w*^*1118*^ on 200 μM MP + variable concentrations of metformin. Lifespans on food without metformin are replicates from Fig 2A (for endo-) and S4A Fig (for endo/trans-) to provide a better comparison with metformin effect. **(E)** Expression of insulin signalling markers 4E-BP, ImpL2 and InR in *tubGS*>mtEcoBI endo/trans- (*UAS-mtHsdM*.*UAS-mtHsdS*/+;*UASmtHsdR* K477R/*tubGS*), endo- (*UAS-mtHsdM*.*UAS-mtHsdS*/+;*UAS-mtHsdR* D298E/*tubGS*) and func (*UAS-mtHsdM*.*UAS-mtHsdS*/+;*UAS-mtHsdR*/*tubGS*) strains, *p*<0.05 (*), *p*<0.01 (**), n = 3.
(PDF)

S11 Fig Effects of adult-onset mtEcoBI expression in muscle tissue. **(A)** Lifespans of flies from *mhcGS*>mtEcoBI endo/trans- (*UAS-mtHsdM*.*UASmtHsdS*/+;*UAS-mtHsdR* K477R/*mhcGS)*, endo- (*UAS-mtHsdM*.*UAS-mtHsdS*/+;*UAS-mtHsdR* D298E/*mhcGS*) and func (*UAS-mtHsdM UAS-mtHsdS*/+;*UAS-mtHsdR*/*mhcGS*) strains kept on food with 200 μM MP. **(B)** BsrGI-digested mtDNA from *mhcGS*>mtEcoBI endo/trans- (*UASmtHsdM*.*UAS-mtHsdS*/+;*UAS-mtHsdR* K477R/*mhcGS)*, endo- (*UAS-mtHsdM*.*UASmtHsdS*/+;*UAS-mtHsdR* D298E/*mhcGS*) and func (*UAS-mtHsdM*.*UAS-mtHsdS*/+;*UASmtHsdR*/*mhcGS*) strains on separate days after induction with 200 μM MP. Red arrow points to major break points. **(C)** Triacylglyceride levels in flies from *mhcGS*>mtEcoBI endo/trans- (*UASmtHsdM*.*UAS-mtHsdS*/+;*UAS-mtHsdR* K477R/*mhcGS)*, endo- (*UAS-mtHsdM*.*UASmtHsdS*/+;*UAS-mtHsdR* D298E/*mhcGS*) and func (*UAS-mtHsdM*.*UAS-mtHsdS*/+;*UASmtHsdR*/*mhcGS*) strains after 9 days of induction with 200 μM MP, *p*<0.05 (*), *p*<0.0001 (****), n = 4. **(D)** Hemolymph glycemia in flies from *mhcGS*>mtEcoBI endo/trans- (*UAS-mtHsdM*.*UAS-mtHsdS*/+;*UAS-mtHsdR* K477R/*mhcGS)*, endo- (*UAS-mtHsdM*.*UAS-mtHsdS*/+;*UAS-mtHsdR* D298E/*mhcGS*) and func (*UAS-mtHsdM*.*UAS-mtHsdS*/+;*UAS-mtHsdR*/*mhcGS*) strains after 9 days of induction with 200 μM MP, n = 3. **(E)** Respiration of *mhcGS*>mtEcoBI endo/trans- (*UAS-mtHsdM*.*UAS-mtHsdS*/+;*UASmtHsdR* K477R/*mhcGS)*, endo- (*UAS-mtHsdM*.*UAS-mtHsdS*/+;*UAS-mtHsdR* D298E/*mhcGS*) and func (*UAS-mtHsdM*.*UAS-mtHsdS*/+;*UAS-mtHsdR*/*mhcGS*) strains after 6 days of induction with 200 μM MP, n = 3–4.
(PDF)

S12 Fig ATP levels and activities of central regulatory glycolytic enzymes in *tubGS*>mtEcoBI strains. **(A)** ATP amount from *tubGS*>mtEcoBI endo/trans- (*UAS-mtHsdM*.*UASmtHsdS*/+;*UAS-mtHsdR* K477R/*tubGS*), endo- (*UAS-mtHsdM*.*UAS-mtHsdS*/+;*UAS-mtHsdR* D298E/*tubGS*) and func (*UAS-mtHsdM*.*UAS-mtHsdS*/+;*UAS-mtHsdR*/*tubGS*) strains 10 days after induction with 200 μM MP, *p*<0.001 (***), n = 5. **(B)** Activities of three rate-limiting glycolytic enzymes from *tubGS*>mtEcoBI endo/trans- (*UAS-mtHsdM*.*UAS-mtHsdS*/+;*UAS-mtHsdR* K477R/*tubGS*), endo- (*UAS-mtHsdM*.*UAS-mtHsdS*/+;*UAS-mtHsdR* D298E/*tubGS*) and func (*UAS-mtHsdM*.*UASmtHsdS*/+;*UAS-mtHsdR*/*tubGS*) strains 8 days after induction on 200 μM MP, n = 3.
(PDF)

S13 Fig Levels of several glycolytic metabolites and intermediates of serine synthesis in *tubGS*>mtEcoBI strains. Levels of glycolysis and serine synthesis intermediates from *tubGS*>mtEcoBI (*UAS-mtHsdM*.*UASmtHsdS*/+;*UAS-mtHsdR* K477R/*tubGS*), endo- (*UAS-mtHsdM*.*UAS-mtHsdS*/+;*UAS-mtHsdR* D298E/*tubGS*) and func (*UAS-mtHsdM*.*UAS-mtHsdS*/+;*UAS-mtHsdR*/*tubGS*) strains 6 days after induction with 200 μM MP, ns–not significant, *p*<0.05(*), *p*<0,01 (**), n = 3–5. Dashed arrows represent more than one reaction between intermediates.
(PDF)

S14 Fig Levels of amino acids Trp, Ile, Leu, Lys, Asp and Glu in *tubGS*>mtEcoBI strains. Concentration of ketogenic amino acids Trp, Ile, Leu, Lys, Asp and Glu in *tubGS*>mtEcoBI endo/trans- (*UAS-mtHsdM*.*UAS-mtHsdS*/+;*UAS-mtHsdR* K477R/*tubGS*), endo- (*UAS-mtHsdM*.*UAS-mtHsdS*/+;*UAS-mtHsdR* D298E/*tubGS*) and func (*UAS-mtHsdM*.*UASmtHsdS*/+;*UAS-mtHsdR*/*tubGS*) strains at day 6 after induction with 200 μM MP, ns–not significant, *p*<0.05 (*), *p*<0.01 (**), *p*<0.0001 (****), n = 5.
(PDF)

S15 Fig Validation of linearity of Ponceau S-signal, western scans used in quantifications and NAD values. **(A)** Densitometry curve of Ponceau S—stained protein signal, data points are mean of two replicates, value represents R^2^. **(B)** Antibody- and Ponceau S-stained western membranes used in quantifications of global acetylation, H3 acetylation (rep1 and rep2 stand for separate technical repeats), mitochondrial acetylation and global PARylation signal from *tubGS*>mtEcoBI endo/trans- (*UASmtHsdM*.*UAS-mtHsdS*/+;*UAS-mtHsdR* K477R/*tubGS*), endo- (*UAS-mtHsdM*.*UASmtHsdS*/+;*UAS-mtHsdR* D298E/*tubGS*) and func (*UAS-mtHsdM*.*UAS-mtHsdS*/+;*UASmtHsdR*/*tubGS*) strains. Quantification areas are shown when less than a full lane was taken for quantifications due to nonspecific signal on acetylated lysine westerns caused by the vicinity of a protein run marker. AI—after induction with 200 μM MP. **(C)** Total NAD and NAD+/NADH ratio in *tubGS*>mtEcoBI endo/trans- (*UAS-mtHsdM*.*UAS-mtHsdS*/+;*UAS-mtHsdR* K477R/*tubGS*), endo- (*UAS-mtHsdM*.*UAS-mtHsdS*/+;*UAS-mtHsdR* D298E/*tubGS*) and func (*UASmtHsdM*.*UAS-mtHsdS*/+;*UAS-mtHsdR*/*tubGS*) strains 10 days after inducton with 200 μM MP, *p*<0.05 (*), *p*<0.0001 (****), n = 4–5.
(PDF)

S16 Fig Comparison of RER between *tubGS*>mtEcoBI strains induced on food without or with citrate supplementation, drawing of cytosolic citrate utilization and lifespans on oxaloacetate-complemented food. **(A)** Side-by-side comparison of RER (data from Figs 3E and 5G) of *tubGS*>mtEcoBI endo/trans- (*UASmtHsdM*.*UAS-mtHsdS*/+;*UAS-mtHsdR* K477R/*tubGS*), endo- (*UAS-mtHsdM*.*UASmtHsdS*/+;*UAS-mtHsdR* D298E/*tubGS*) and func (*UAS-mtHsdM*.*UAS-mtHsdS*/+;*UASmtHsdR*/*tubGS*) strains kept on regular food with 200 μM MP or citrate-supplemented food with 200 μM MP on day 6 after induction, ns-not significant, *p*<0.05 (*). **(B)** Lifespans *tubGS*>mtEcoBI endo/trans- (*UAS-mtHsdM*.*UAS-mtHsdS*/+;*UAS-mtHsdR* K477R/*tubGS*), endo- (*UASmtHsdM*.*UAS-mtHsdS*/+;*UAS-mtHsdR* D298E/*tubGS*) and func (*UAS-mtHsdM*.*UASmtHsdS*/+;*UAS-mtHsdR*/*tubGS*) strains on food containg 200 μM MP with or without 20 mM oxaloacetate. Lifespans on food without oxaloacetate are replicates from Fig 2A to provide a better comparison with oxaloacetate effect. **(C)** Schematic representation of conversion of cytosolic citrate to pyruvate, PDH—pyruvate dehydrogenase.
(PDF)
