# Supplementary material for: Manipulating mtDNA in vivo reprograms metabolism via novel response mechanisms
Source: PLoS Genet. 2019 Oct 4;15(10):e1008410. doi: 10.1371/journal.pgen.1008410 (PMC6795474; doi:10.1371/journal.pgen.1008410)
Supplement: S1 Table — (PDF) [file pgen.1008410.s001.pdf]

Oligonucleotide name	Oligonucleotide sequence
RPL32 for	TGTGCACCAGGAACTTCTTGAA
RPL32 rev	AGGCCCAAGATCGTGAAGAA
ND5 for	GGGTGAGATGGTTTAGGACTTG
ND5 rev	AAGCTACATCCCCAATTCGAT
Hsp 103	GAATTCGGGCAGCAGAACAC
Hsp 105	GCTGACCAAGACGAAAGGAG
Lon3	GGGGTCCATGAAAGCACAAG
Lon5	ACCGAAAATCTCTGCCTGGA
ClpX3	TGAACACCCCGAATGTCTGA
ClpX5	TGGGTCTTGATGAGGTGGAC
4E-BP3	TTCCCCTCAGCAAGCAACTG
4E-BP5	CTCCTGGAGGCACCAAACTTATC
Hsc70-5	TCAGCTTCAGGTTCATGTGC
Hsc70-5	GGAATTGATATCCGCAAGGA
ImpL2-3	AGGTATCGGCGGTATCCTTT
Impl2-5	CCGAGATCACCTGGTTGAAT
InR3	GCAGGAAGCCCTCGATGA
InR5	ACAAAATGTAAAACCTTGCAAATCC
CytB For	GAAAATTCCGAGGGATTCAA
CytB Rev	AACTGGTCGAGCTCCAATTC
Cox2 for	AAAGTTGACGGTACACCTGGA
Cox2 rev	TGATTAGCTCCACAGATTTC
HsdMF	GGACAGGAAGCGGAATACCT
HsdMR	CTGTTTCGGCTGGGTAATGG
HsdSF	CCTACGCCGACTCCATAGAA
HsdSR	CGCTGTTTTCTCCGCTGATT

HsdRF	CCCCTTCTGCTACTCAACCA
HsdRR	GCTTTCGACATATTGCGGGT
