# Supplementary material for: Manipulating mtDNA in vivo reprograms metabolism via novel response mechanisms
Source: PLoS Genet. 2019 Oct 4;15(10):e1008410. doi: 10.1371/journal.pgen.1008410 (PMC6795474; doi:10.1371/journal.pgen.1008410)
Supplement: S2 Table — (PDF) [file pgen.1008410.s002.pdf]

Antibody	Source	Incubation buffer	Prim. dilution	Sec.dilution
HsdR	Abmart	5% milk in PBS + 0,05% Tween	1 : 1 000	1 : 5 000
NDUFS3	Abcam (ab14711)	5% milk in PBS + 0,05% Tween	1 : 20 000	1 : 5 000
porin	Abcam (ab14734)	5% milk in PBS + 0,05% Tween	1 : 5 000	1 : 10 000
Hsp60	Cell Signaling (#4870)	5% milk in PBS + 0,05% Tween	1 : 1 000	1 : 5 000
phospho-Akt	Cell Signaling (#4054)	2,5% milk in PBS + 0,05% Tween	1 : 5 000	1 : 10 000
Akt	Cell Signaling (#9272)	2,5% milk in PBS + 0,05% Tween	1 : 5 000	1 : 10 000
eIF2α	Abcam (ab26197)	5% BSA in TBS + 0,1% Tween	1 : 40 000	1 : 10 000
phospho-eIF2α	Abcam (ab32157)	5% BSA in TBS + 0,1% Tween	1 : 25 000	1 : 10 000
Acetylated lysine	Cell Signaling (#9441)	5% BSA in TBS + 0,1% Tween	1 : 5 000	1 : 10 000
pan-histone 3	Abcam (ab1791)	5% BSA in TBS + 0,1% Tween	1 : 5 000	1 : 10 000
Acetylated histone 3	Abcam (ab47915)	5% BSA in TBS + 0,1% Tween	1 : 5 000	1 : 10 000
Poly-(ADP) ribosylation	Abcam (ab14459)	5% BSA in TBS + 0,1% Tween	1 : 5 000	1 : 10 000
