# Supplementary material for: Manipulating mtDNA in vivo reprograms metabolism via novel response mechanisms
Source: PLoS Genet. 2019 Oct 4;15(10):e1008410. doi: 10.1371/journal.pgen.1008410 (PMC6795474; doi:10.1371/journal.pgen.1008410)
Supplement: S1 Text — (PDF) [file pgen.1008410.s003.pdf]

1. Schmid MR, Anderl I, Vo HT, Valanne S, Yang H, Kronhamn J, et al. Genetic screen in Drosophila larvae links ird1 function to Toll signaling in the fat body and hemocyte motility. PLoS One. 2016;11(7):e0159473.
