# Supplementary figures and images for: Manipulating mtDNA in vivo reprograms metabolism via novel response mechanisms
Source: PLoS Genet. 2019 Oct 4;15(10):e1008410. doi: 10.1371/journal.pgen.1008410 (PMC6795474; doi:10.1371/journal.pgen.1008410)

**A***tubGS>mtMTase*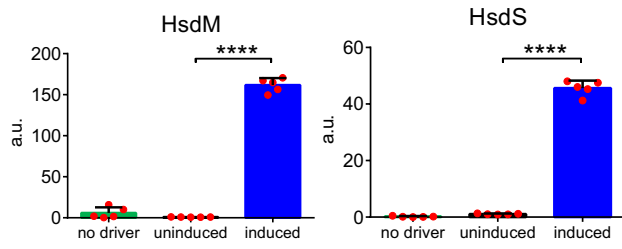*tubGS>mtEcoBI isoforms*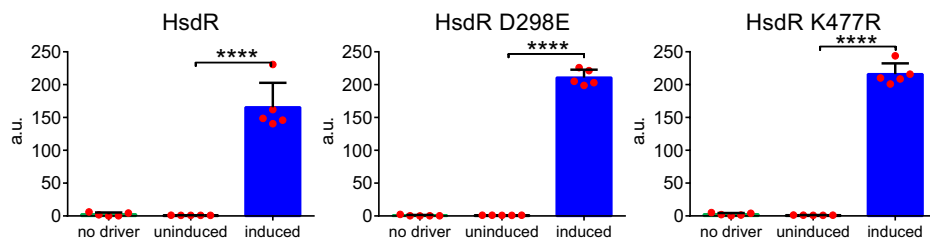**C**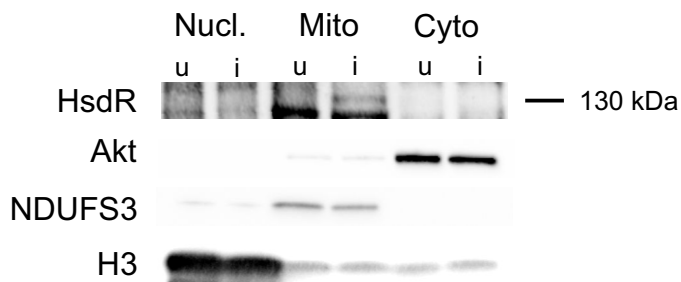**B**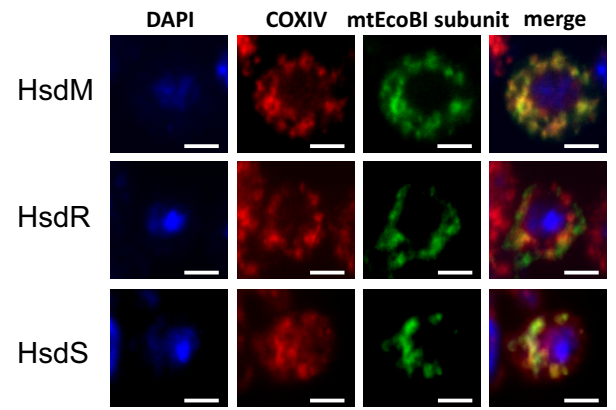**D**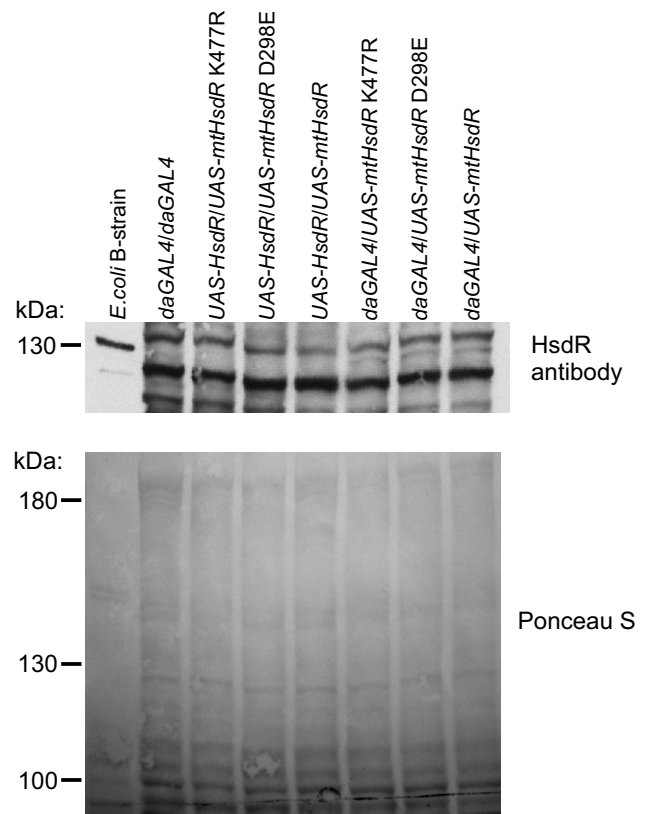

Supplement: S1 Fig — (A) Steady-state mRNA levels of HsdM, HsdS and HsdR subunits after induction with 200 μM mifepristone for 6 days either in strain carrying the methyltransferase-capable form of mtEcoBI consisting only of HsdM and HsdS subunits (UAS-mtHsdM.UAS-mtHsdS/+;tubGS/+) or in strains carrying the full enzyme isoforms (UAS-mtHsdM.UAS-mtHsdS/+;UAS-mtHsdR/tubGS, UASmtHsdM.UAS-mtHsdS/+;UAS-mtHsdR D298E/tubGS, UAS-mtHsdM.UAS-mtHsdS/+;UAS-mtHsdR K477R/tubGS). p<0.0001 (****), n = 5. (B) Subcellular localisation of HsdS fused to citrate synthase MTS and to V5-epitope in transiently expressing S2 cells. Cells were stained for DAPI and labeled with antibodies against COXIVand V5-epitope, followed by incubation with secondary antibodies conjugated with Alexa 568 (green) and Alexa 488 (red) respectively. Scale bar is 5 mm. (C) Subcellular fractionation of Drosophila tissue from UAS-mtHsdM.UAS-mtHsdS/+;UAS-mtHsdR/tubGS strain after 6 days of incubation either on regular or 200 μM mifepristone-containing food using HsdR, Akt (cytosolic marker), NDUFS3 (mitochondrial marker) and histone 3 (nuclear marker) antibodies, u—uninduced, i—induced. (D) Western of Drosophila strains expressing different isoforms of HsdR subunit with antibodies against HsdR, E.coli B-strain served as a control. Ponceau S-stained membrane was used as a loading control. (PDF) [file pgen.1008410.s005.pdf]

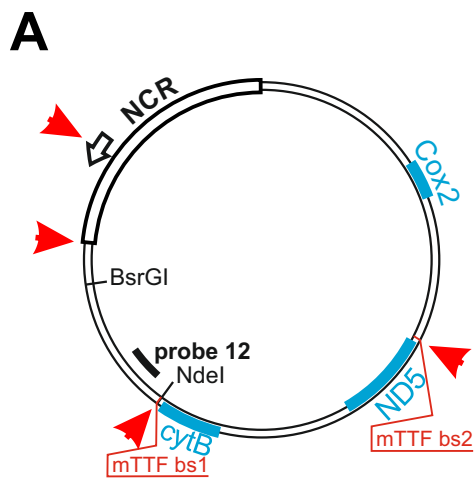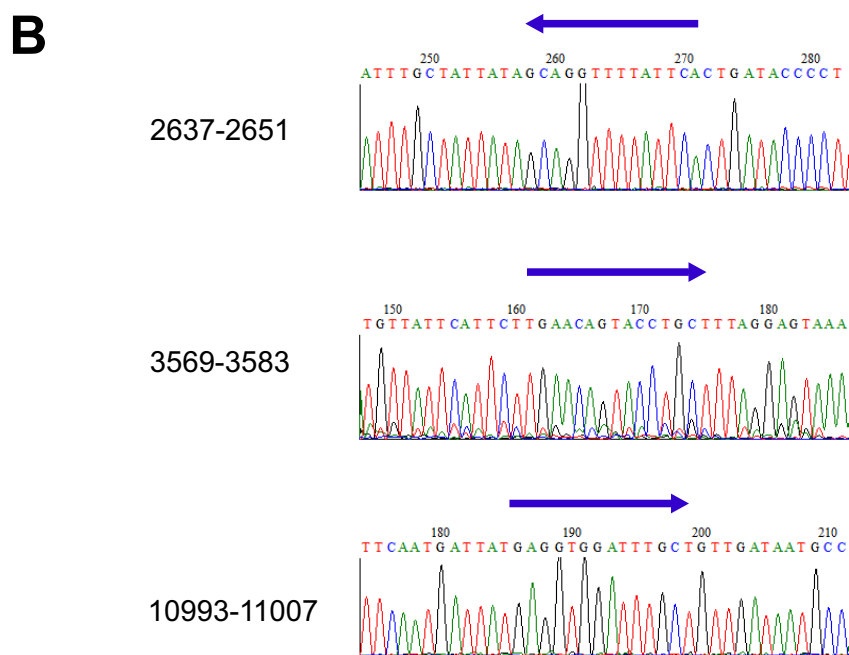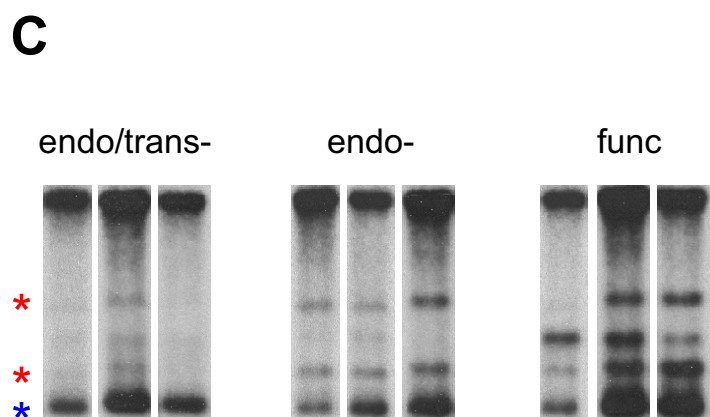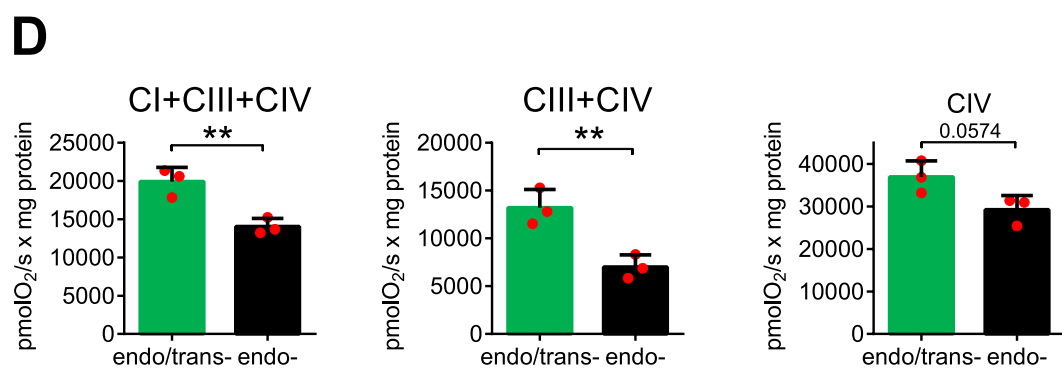

E

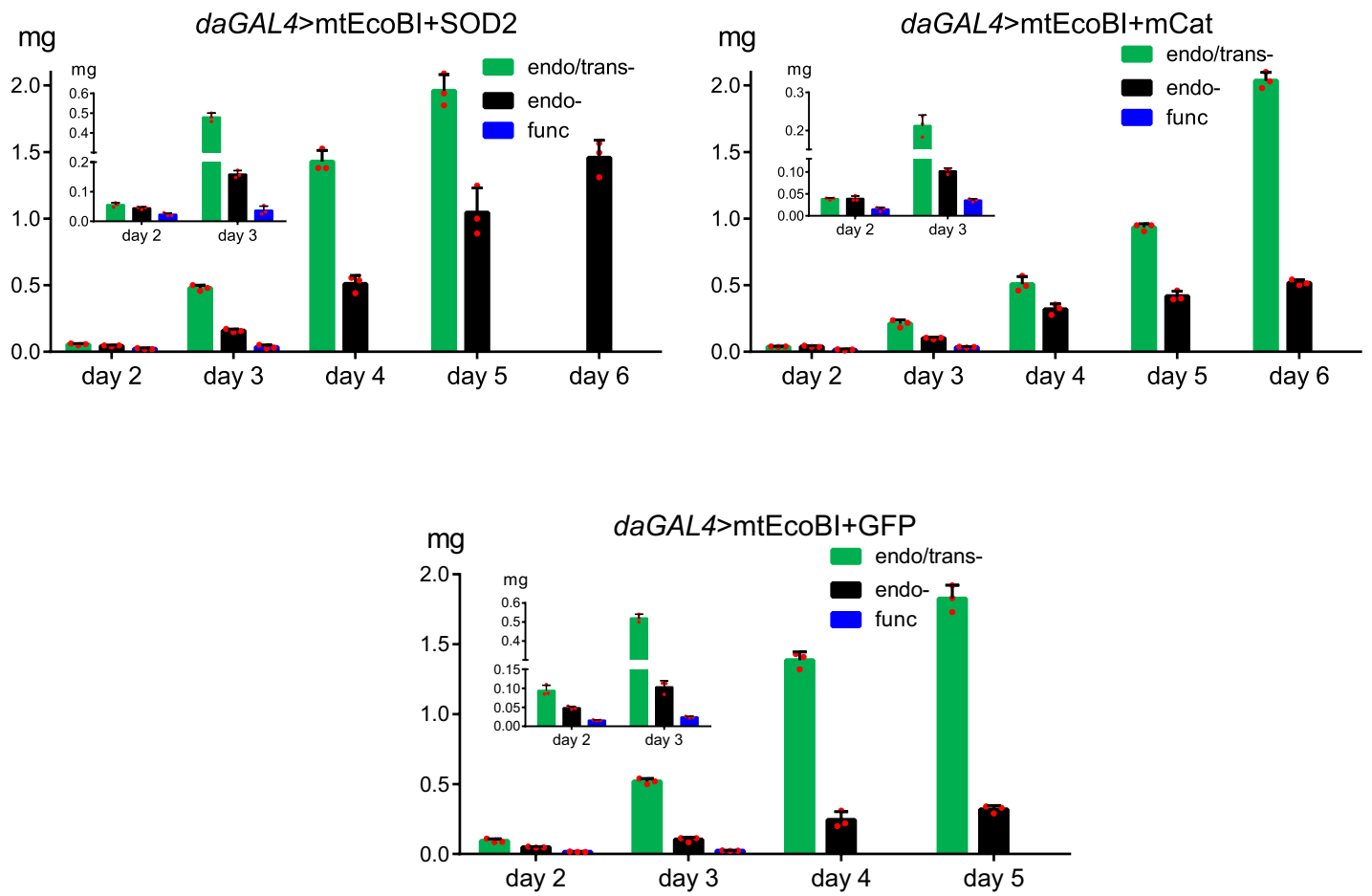

Supplement: S2 Fig — (A) Schematic map of Drosophila mtDNA showing major mtEcoBI cleavage sites (red arrows), positions of mTTF/mTERF5 binding sites (mTTF bs1 and bs2), positions of genes used for transcript measurements (in blue), non-coding region (NCR) and origin of replication (arrow within NCR). (B) Sequences of mtDNA regions containing mtEcoBI binding sites TGA-(N)8-TGCT (blue arrows) in strain UAS-mtHsdM.UAS-mtHsdS/+;UAS-mtHsdR/daGAL4 3 days after egg laying. Numbers refer to position of nucleotides in D. melanogaster mtDNA in NCBI nucleotide databank entry NC_001709. (C) 1D gel electrophoresis of uncut mtDNA samples from larvae of endo/trans- (UAS-mtHsdM.UAS-mtHsdS/+;UAS-mtHsdR K477R/daGAL4), endo- (UASmtHsdM.UAS-mtHsdS/+;UAS-mtHsdR D298E/daGAL4) and func (UAS-mtHsdM.UASmtHsdS/+;UAS-mtHsdR/daGAL4) strains used for quantifications of covalently closed (cc) forms of different linking number in Fig 1D. (D) State III respiration of mitochondria isolated from UASmtHsdM.UAS-mtHsdS/+;UAS-mtHsdR/daGAL4, UAS-mtHsdM.UAS-mtHsdS/+;UAS-mtHsdR D298E/daGAL4, UAS-mtHsdM.UAS-mtHsdS/+;UAS-mtHsdR K477R/daGAL4 strain larvae day 2 AEL, p<0.01 (**), n = 3. (E) Wet weight of UAS-mtHsdM.UAS-mtHsdS/UAS-SOD2;UAS-mtHsdR/daGAL4, UASmtHsdM.UAS-mtHsdS/UAS-SOD2;UAS-mtHsdR D298E/daGAL4, UAS-mtHsdM.UASmtHsdS/ UAS-SOD2;UAS-mtHsdR K477R/daGAL4 and UAS-mtHsdM.UAS-mtHsdS/UASmCat; UAS-mtHsdR/daGAL4, UAS-mtHsdM.UAS-mtHsdS/UAS-mCat;UAS-mtHsdR D298E/daGAL4, UAS-mtHsdM.UAS-mtHsdS/UAS-mCat;UAS-mtHsdR K477R/daGAL4 and UAS-mtHsdM.UASmtHsdS/UAS-GFP;UAS-mtHsdR/daGAL4, UAS-mtHsdM.UAS-mtHsdS/UAS-GFP;UAS-mtHsdR D298E/daGAL4, UAS-mtHsdM.UAS-mtHsdS/UAS-GFP;UAS-mtHsdR K477R/daGAL4 larvae. Days mark time after egg laying, n = 3. (PDF) [file pgen.1008410.s006.pdf]

**A**

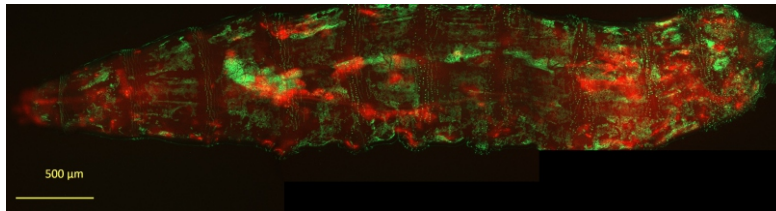

endo-

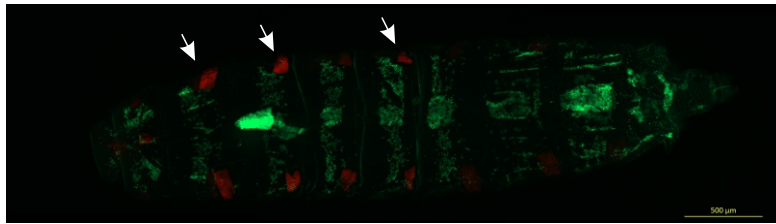

endo/trans-

**B**

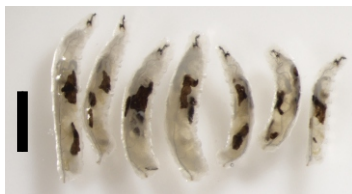

+ 1,5 mM NAC

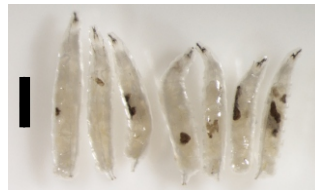

SOD2 overexpression

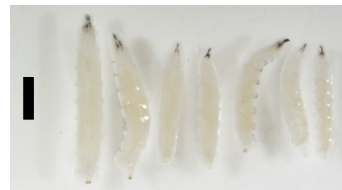

Supplement: S3 Fig — (A) Microscopy of mo-mCherry eater-MSNF9 GFP/FM7a;UAS-mtHsdM UASmtHsdS/+;UAS-mtHsdR D298E/daGAL4 (endo-) and MSNF9mo-mCherry eater-GFP/FM7a;UASmtHsdM.UAS-mtHsdS/+;UAS-mtHsdR K477R/daGAL4 (endo/trans-) L3 larvae (5 days after egg laying) showing green plasmatocytes and red lamellocyte signal. White arrows point to the red signalin larval muscle that is caused by labeling artefact of the given reporter system [1]. Scale bar is 0,5 mm. (B) UAS-mtHsdM.UAS-mtHsdS/+;UAS-mtHsdR D298E/daGAL4 (endo-) larvae of L3 stage (5 days after egg laying) showing melanotic nodules reared on regular food (left) and on food supplemented with 1,5 mM N-acetyl cysteine. „SOD2 overexpression” refers to UAS-mtHsdM.UASmtHsdS/UAS-SOD2;UAS-mtHsdR D298E/daGAL4 (endo-) larvae grown on regular food. Scale bar is 1 mm. (PDF) [file pgen.1008410.s007.pdf]

**A**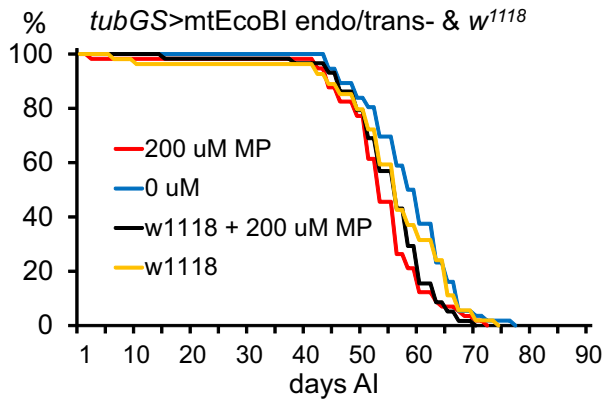**B**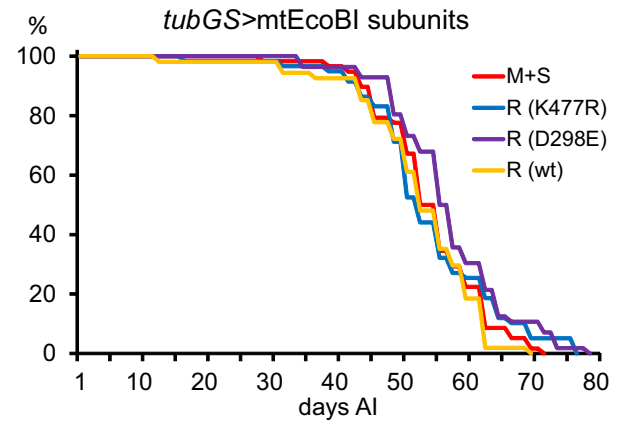**C**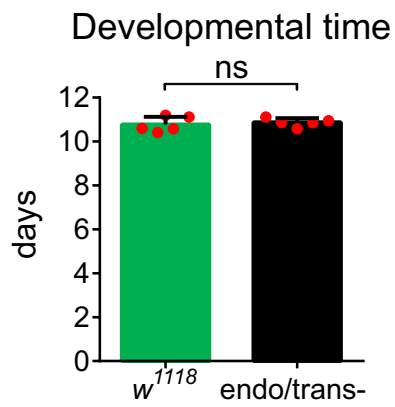**D**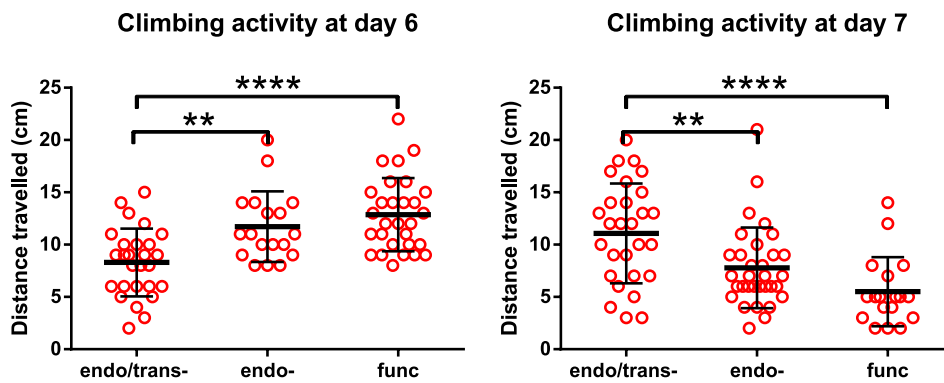

E

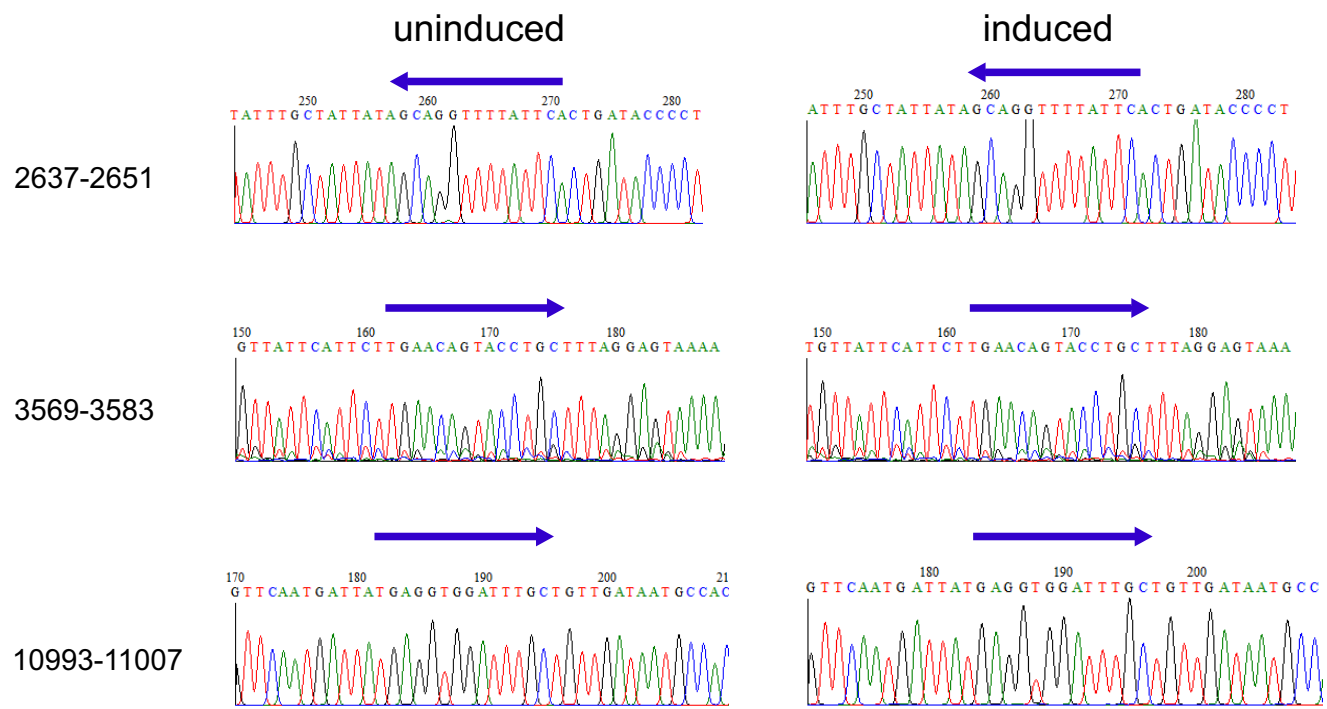

Supplement: S4 Fig — (A) Lifespans of tubGS>mtEcoBI endo/trans- (UAS-mtHsdM.UAS-mtHsdS/+;UASmtHsdR K477R/tubGS) and w1118 strains on food with and without 200 μM mifepristone. (B) Lifespans of strains expressing different combinations of mtEcoBI subunits from tubGS driver (M+S: UAS-mtHsdM.UAS-mtHsdS/+;tubGS/+, R (K477R): UAS-mtHsdR/tubGS, R (D298E): UAS-mtHsdR D298E/tubGS, R (wt): UAS-mtHsdR K477R/tubGS). (C) Developmental time comparison between w1118 and endo/trans-, ns–not significant, n = 5. (D) Climbing activities of flies from tubGS>mtEcoBI strains at days 6 and 7 after induction with 200 μM mifepristone, p<0.01 (**), p<0.0001 (****), n = 18–32. (E) Sequences of mtDNA regions containing mtEcoBI binding sites TGA-(N) -TGCT (blue arrows) in tubGS>mtEcoBI func strain (UAS-mtHsdM.UAS-mtHsdS/+;UAS-mtHsdR/tubGS) with or without induction with 200 μM MP for 10 days. Numbers refer to position of nucleotides in D. melanogaster mtDNA in NCBI nucleotide databank entry NC_001709. (PDF) [file pgen.1008410.s008.pdf]

**A**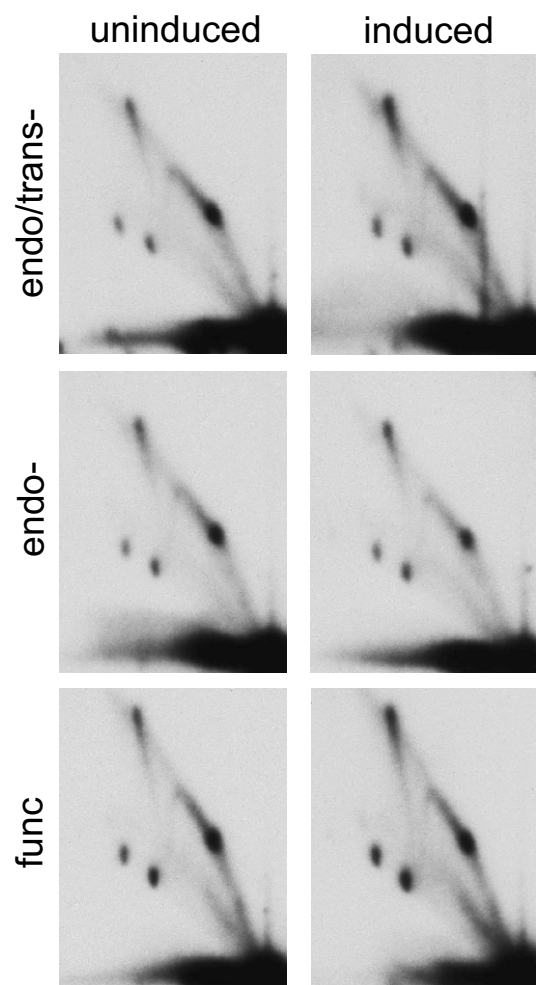**B**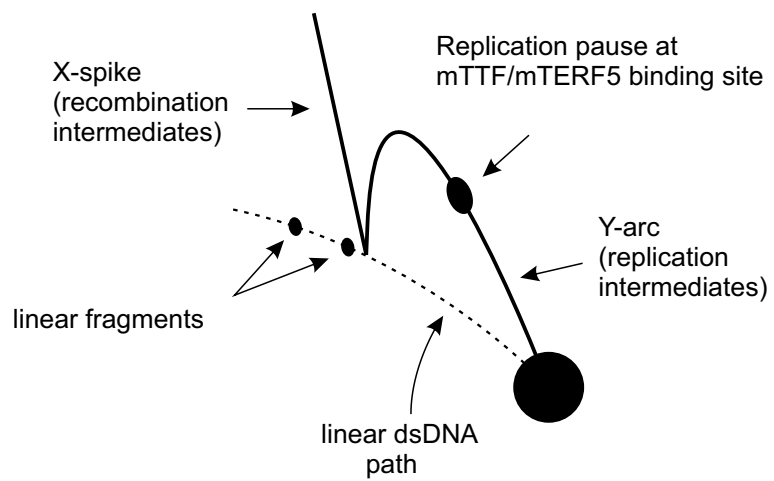

Supplement: S5 Fig — (A) 2DNAGE of mtDNA Cla fragment (nt 7874–12951 in NC_001709) from tubGS>mtEcoBI endo/trans- ((UAS-mtHsdM.UAS-mtHsdS/+;UAS-mtHsdR K477R/tubGS), endo- (UASmtHsdM.UAS-mtHsdS/+;UAS-mtHsdR D298E/tubGS) and func (UAS-mtHsdM.UAS-mtHsdS/+;UASmtHsdR/tubGS) strains kept 10 days on food with or without 200 μM MP (induced/uninduced). (B) Drawing detailing major replication and recombination intermediates separated on 2DNAGE on panel A. (PDF) [file pgen.1008410.s009.pdf]

NDUFS3

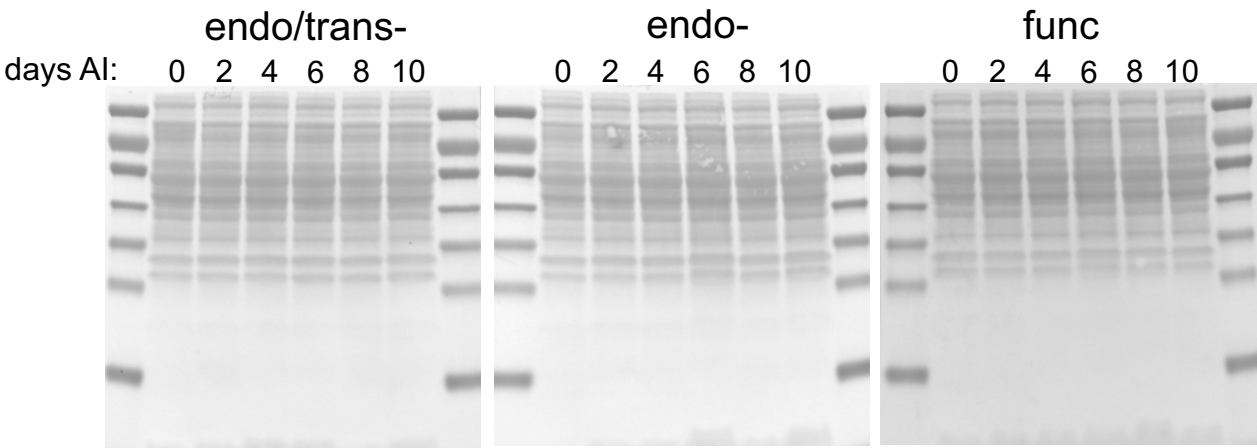

porin

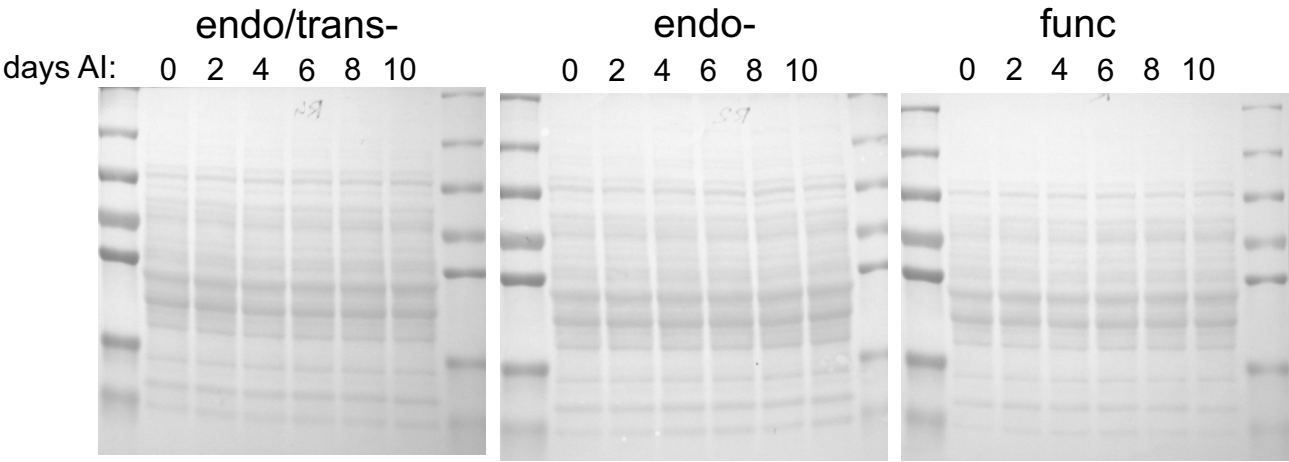

Supplement: S6 Fig — Total protein amount visualized with Ponceau S—staining from tubGS>mtEcoBI. endo/trans- (UAS-mtHsdM UAS-mtHsdS/+;UAS-mtHsdR K477R/tubGS), endo- (UAS-mtHsdM.UAS-mtHsdS/+;UAS-mtHsdR D298E/tubGS) and func (UAS-mtHsdM UAS-mtHsdS/+;UASmtHsdR/tubGS) strains. AI: after induction on 200 μM MP. (PDF) [file pgen.1008410.s010.pdf]

**A**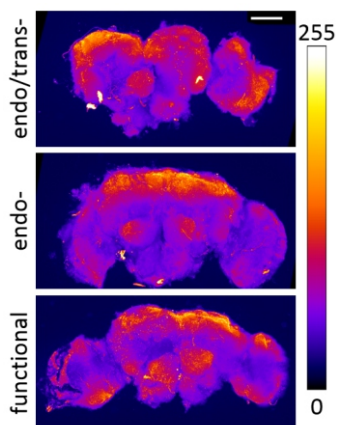**B**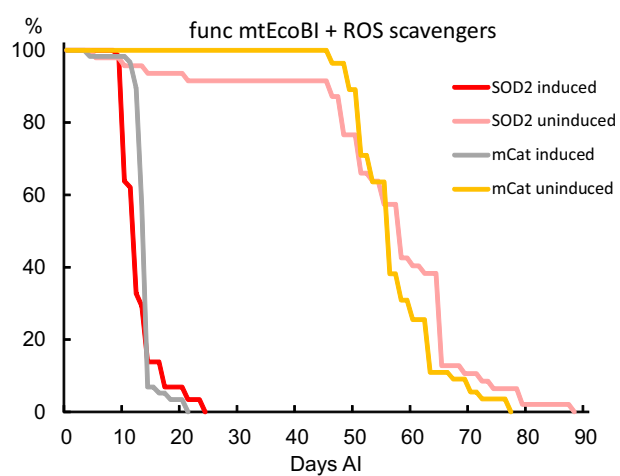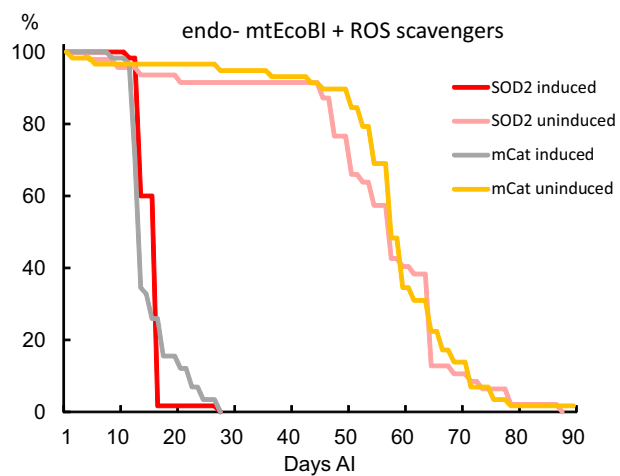

Supplement: S7 Fig — (A) Maximum projections of dihydroethidium (DHE)-stained brains from indicated strains after 6 days of induction with 200 μM MP. Scale bar 100 μm. (B) Lifespans of indicated strains with co-overexpression of either SOD2 (UAS-mtHsdM.UAS-mtHsdS/UAS-SOD2;UASmtHsdR/tubGS and UAS-mtHsdM.UAS-mtHsdS/UAS-SOD2;UAS-mtHsdR D298E/tubGS) or mCat (UAS-mtHsdM.UAS-mtHsdS/UAS-mCat;UAS-mtHsdR/tubGS and UAS-mtHsdM.UASmtHsdS/UAS-mCat;UAS-mtHsdR D298E/tubGS). (PDF) [file pgen.1008410.s011.pdf]

**A**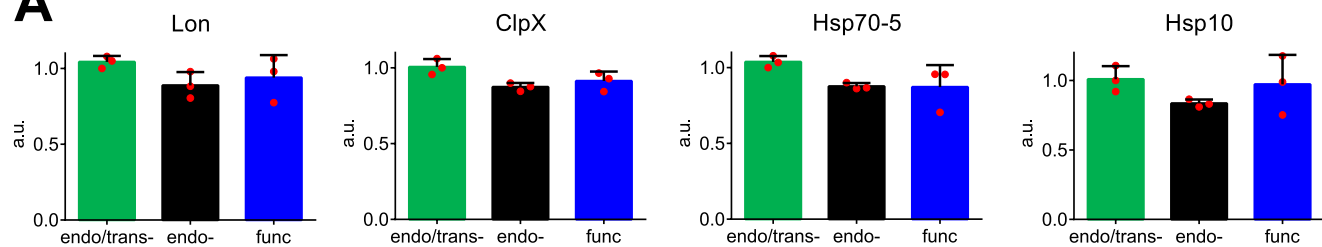**B**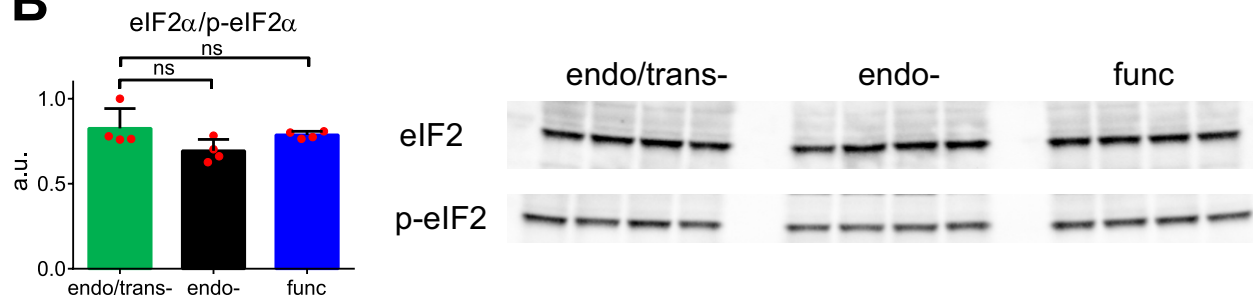

Supplement: S8 Fig — (A) Steady-state mRNA levels of several mtUPR markers from tubGS>mtEcoBI endo/trans- (UAS-mtHsdM.UAS-mtHsdS/+;UAS-mtHsdR K477R/tubGS), endo- (UASmtHsdM.UAS-mtHsdS/+;UAS-mtHsdR D298E/tubGS) and func (UAS-mtHsdM.UASmtHsdS/+;UAS-mtHsdR/tubGS) strains after 10 days of induction with 200 μM MP, n = 3. (B) Ratio of total eIF2a to phosphorylated eIF2a from tubGS>mtEcoBI endo/trans- ((UAS-mtHsdM.UASmtHsdS/+;UAS-mtHsdR K477R/tubGS), endo- (UAS-mtHsdM.UAS-mtHsdS/+;UAS-mtHsdR D298E/tubGS) and func (UAS-mtHsdM.UAS-mtHsdS/+;UAS-mtHsdR/tubGS) strains after 10 days of induction with 200 μM MP, ns—not significant, n = 4. Westerns used for quantifications are shown on the right. (PDF) [file pgen.1008410.s012.pdf]

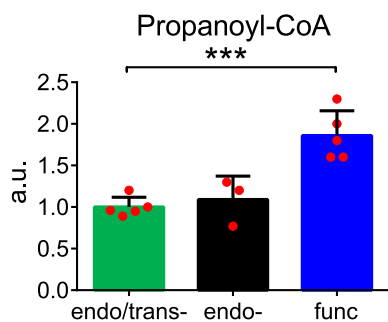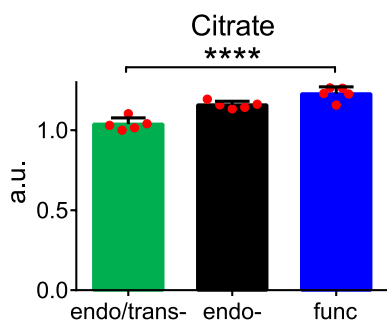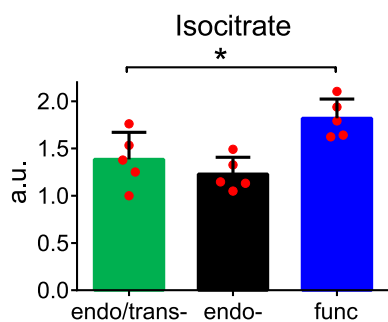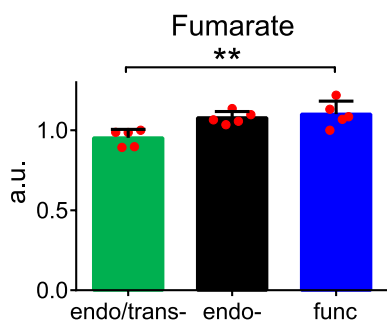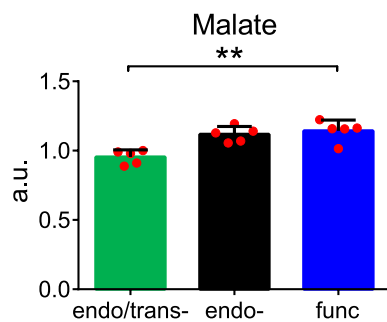

Supplement: S9 Fig — Levels of propanoyl-CoA and TCA intermediates from tubGS>mtEcoBI endo/trans-(UAS-mtHsdM.UAS-mtHsdS/+;UAS-mtHsdR K477R/tubGS), endo- (UAS-mtHsdM.UASmtHsdS/+;UAS-mtHsdR D298E/tubGS) and func (UAS-mtHsdM.UAS-mtHsdS/+;UASmtHsdR/tubGS) strains after 6 days of induction with 200 μM MP., p<0.05(*), p<0.01 (**), p<0.001 (***), p<0.0001 (****), n = 3 or 5. (PDF) [file pgen.1008410.s013.pdf]

**A**TAG in *tubGS>MS* strain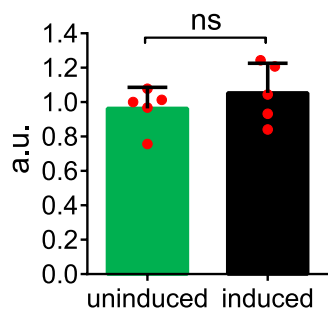TAG in *w<sup>1118</sup>* vs endo/trans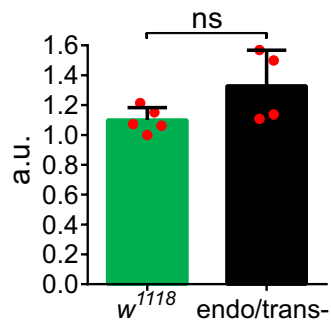**B**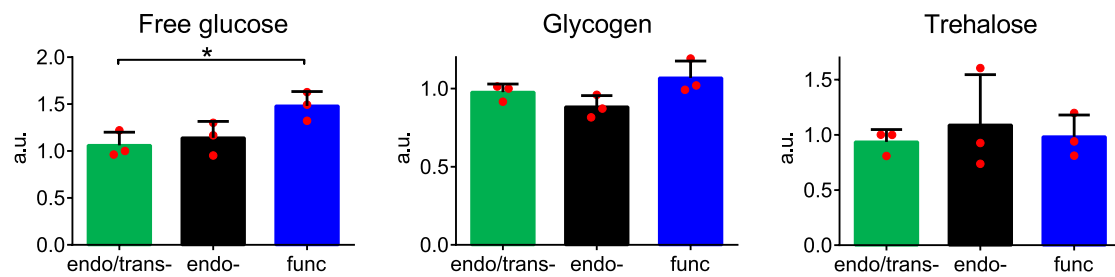**C**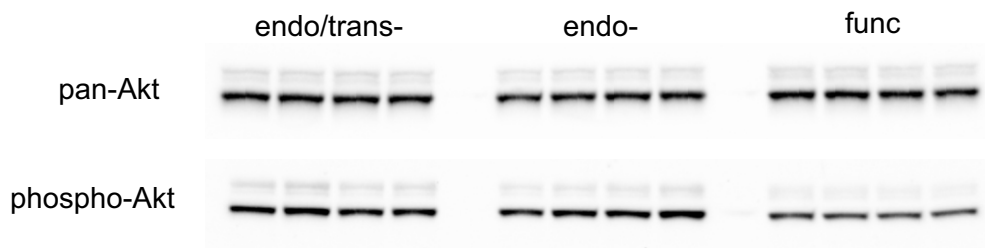**D**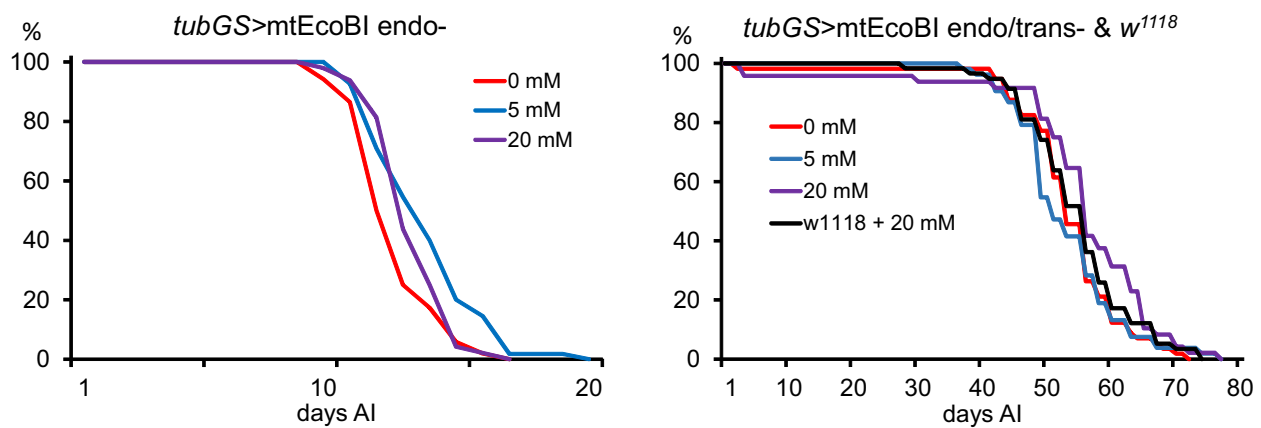

E

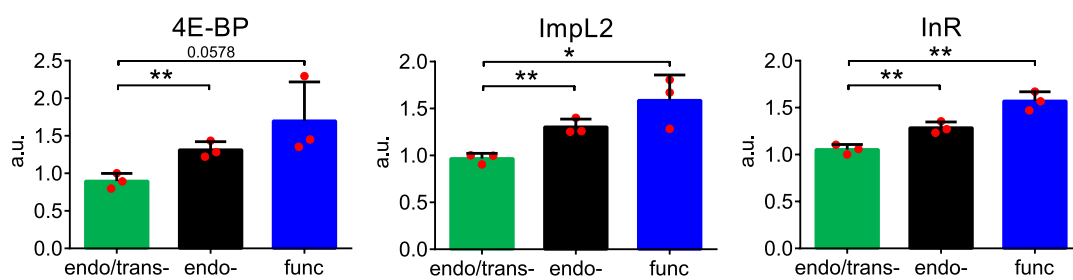

Supplement: S10 Fig — (A) Comparison on TAG between age-matched tubGS>MTase ((UAS-mtHsdM.UASmtHsdS/+;tubGS/+) strains and between w1118 and tubGS>mtEcoBI endo/trans- (mtHsdS/+;UAS-mtHsdR K477R/tubGS) strains after 10 days either on regular or MP-supplemented (200 μM) food, ns–not significant, n = 5. (B) Levels of carbohydrates from tubGS>mtEcoBI endo/trans- (UAS-mtHsdM.UAS-mtHsdS/+;UAS-mtHsdR K477R/tubGS), endo- (UAS-mtHsdM.UASmtHsdS/+;UAS-mtHsdR D298E/tubGS) and func (UAS-mtHsdM.UAS-mtHsdS/+;UASmtHsdR/tubGS) strains (normalized to protein content) after 6 days of induction with 200 μM MP, p<0.05 (*), n = 5. (C) Westerns of tubGS>mtEcoBI endo/trans- ((UAS-mtHsdM.UAS-mtHsdS/+;UAS-mtHsdR K477R/tubGS), endo- (UAS-mtHsdM.UAS-mtHsdS/+;UAS-mtHsdR D298E/tubGS) and func (UAS-mtHsdM.UAS-mtHsdS/+;UAS-mtHsdR/tubGS) flies with pan-Akt and phospho-Akt antibodies after 10 days of induction with 200 μM MP used in quantifications shown in Fig 3H. (D) Lifespans of tubGS>mtEcoBI endo- (UAS-mtHsdM.UAS-mtHsdS/+;UAS-mtHsdR D298E/tubGS) and endo/trans- (UAS-mtHsdM.UAS-mtHsdS/+;UAS-mtHsdR K477R/tubGS) strains with w1118 on 200 μM MP + variable concentrations of metformin. Lifespans on food without metformin are replicates from Fig 2A (for endo-) and S4A Fig (for endo/trans-) to provide a better comparison with metformin effect. (E) Expression of insulin signalling markers 4E-BP, ImpL2 and InR in tubGS>mtEcoBI endo/trans- (UAS-mtHsdM.UAS-mtHsdS/+;UASmtHsdR K477R/tubGS), endo- (UAS-mtHsdM.UAS-mtHsdS/+;UAS-mtHsdR D298E/tubGS) and func (UAS-mtHsdM.UAS-mtHsdS/+;UAS-mtHsdR/tubGS) strains, p<0.05 (*), p<0.01 (**), n = 3. (PDF) [file pgen.1008410.s014.pdf]

**A**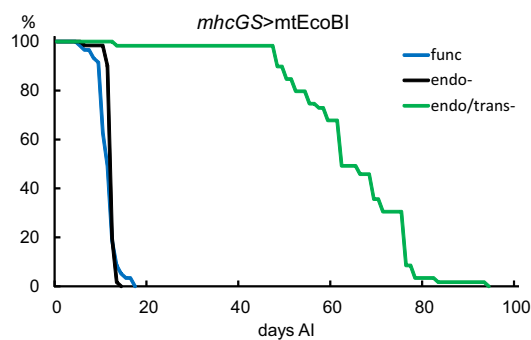**B**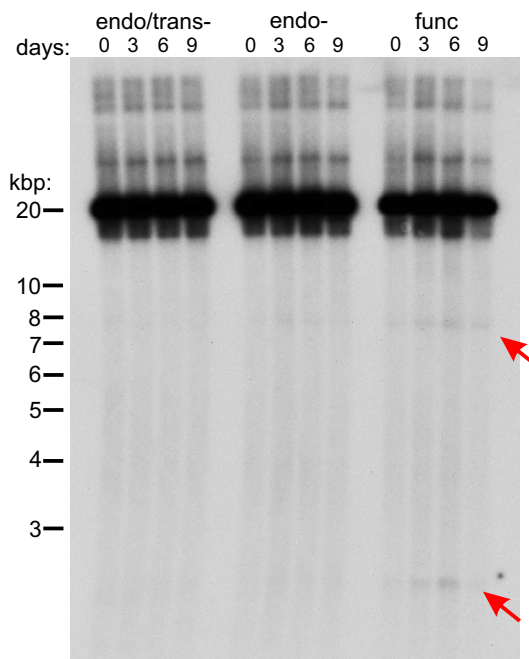**C**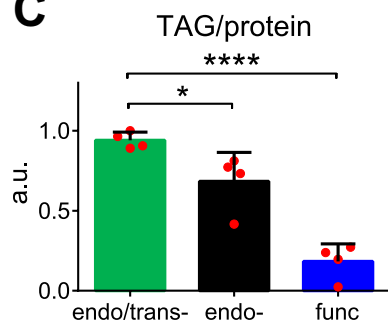**D**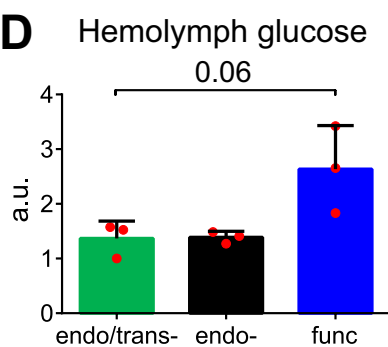**E**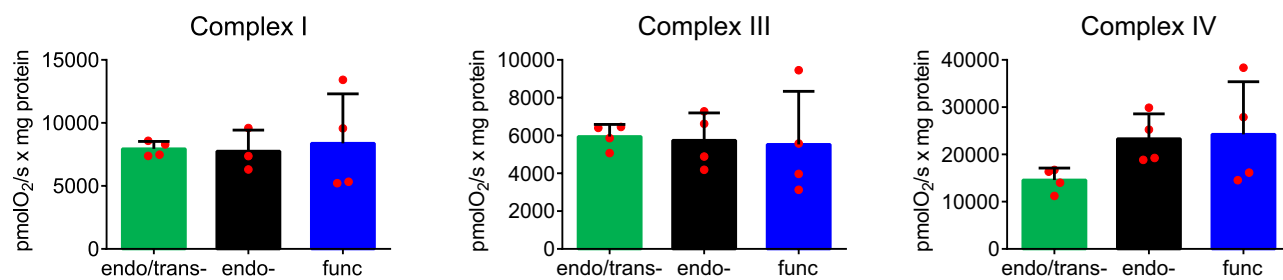

Supplement: S11 Fig — (A) Lifespans of flies from mhcGS>mtEcoBI endo/trans- (UAS-mtHsdM.UASmtHsdS/+;UAS-mtHsdR K477R/mhcGS), endo- (UAS-mtHsdM.UAS-mtHsdS/+;UAS-mtHsdR D298E/mhcGS) and func (UAS-mtHsdM UAS-mtHsdS/+;UAS-mtHsdR/mhcGS) strains kept on food with 200 μM MP. (B) BsrGI-digested mtDNA from mhcGS>mtEcoBI endo/trans- (UASmtHsdM.UAS-mtHsdS/+;UAS-mtHsdR K477R/mhcGS), endo- (UAS-mtHsdM.UASmtHsdS/+;UAS-mtHsdR D298E/mhcGS) and func (UAS-mtHsdM.UAS-mtHsdS/+;UASmtHsdR/mhcGS) strains on separate days after induction with 200 μM MP. Red arrow points to major break points. (C) Triacylglyceride levels in flies from mhcGS>mtEcoBI endo/trans- (UASmtHsdM.UAS-mtHsdS/+;UAS-mtHsdR K477R/mhcGS), endo- (UAS-mtHsdM.UASmtHsdS/+;UAS-mtHsdR D298E/mhcGS) and func (UAS-mtHsdM.UAS-mtHsdS/+;UASmtHsdR/mhcGS) strains after 9 days of induction with 200 μM MP, p<0.05 (*), p<0.0001 (****), n = 4. (D) Hemolymph glycemia in flies from mhcGS>mtEcoBI endo/trans- (UAS-mtHsdM.UAS-mtHsdS/+;UAS-mtHsdR K477R/mhcGS), endo- (UAS-mtHsdM.UAS-mtHsdS/+;UAS-mtHsdR D298E/mhcGS) and func (UAS-mtHsdM.UAS-mtHsdS/+;UAS-mtHsdR/mhcGS) strains after 9 days of induction with 200 μM MP, n = 3. (E) Respiration of mhcGS>mtEcoBI endo/trans- (UAS-mtHsdM.UAS-mtHsdS/+;UASmtHsdR K477R/mhcGS), endo- (UAS-mtHsdM.UAS-mtHsdS/+;UAS-mtHsdR D298E/mhcGS) and func (UAS-mtHsdM.UAS-mtHsdS/+;UAS-mtHsdR/mhcGS) strains after 6 days of induction with 200 μM MP, n = 3–4. (PDF) [file pgen.1008410.s015.pdf]

**A**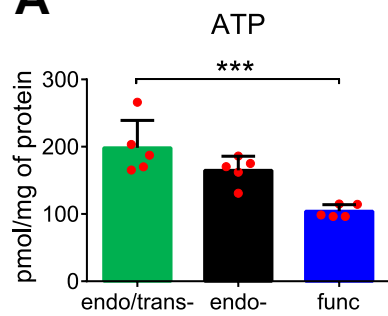**B**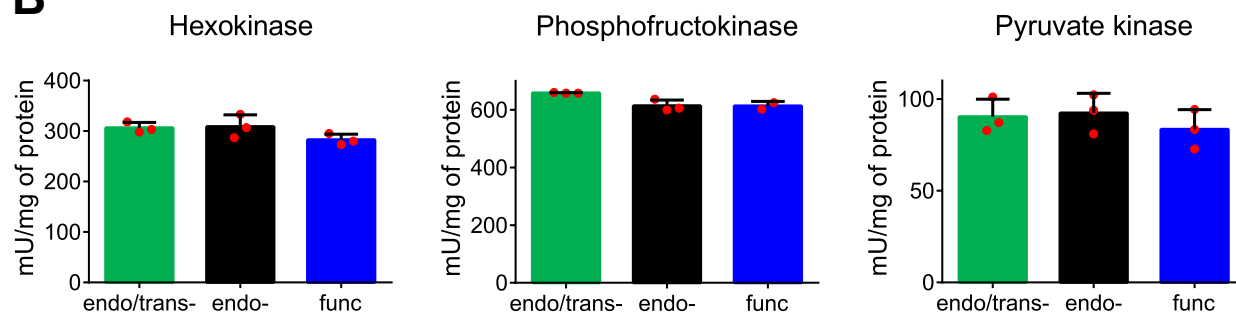

Supplement: S12 Fig — (A) ATP amount from tubGS>mtEcoBI endo/trans- (UAS-mtHsdM.UASmtHsdS/+;UAS-mtHsdR K477R/tubGS), endo- (UAS-mtHsdM.UAS-mtHsdS/+;UAS-mtHsdR D298E/tubGS) and func (UAS-mtHsdM.UAS-mtHsdS/+;UAS-mtHsdR/tubGS) strains 10 days after induction with 200 μM MP, p<0.001 (***), n = 5. (B) Activities of three rate-limiting glycolytic enzymes from tubGS>mtEcoBI endo/trans- (UAS-mtHsdM.UAS-mtHsdS/+;UAS-mtHsdR K477R/tubGS), endo- (UAS-mtHsdM.UAS-mtHsdS/+;UAS-mtHsdR D298E/tubGS) and func (UAS-mtHsdM.UASmtHsdS/+;UAS-mtHsdR/tubGS) strains 8 days after induction on 200 μM MP, n = 3. (PDF) [file pgen.1008410.s016.pdf]

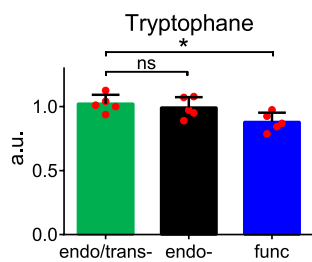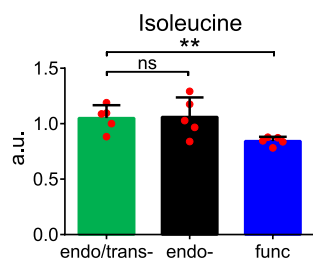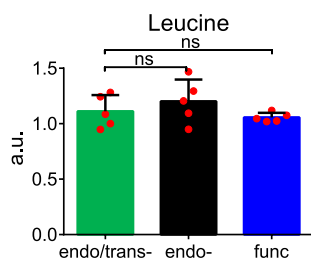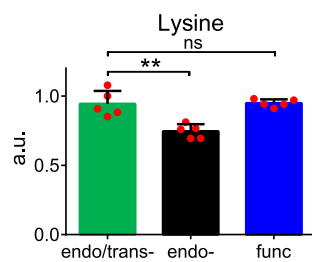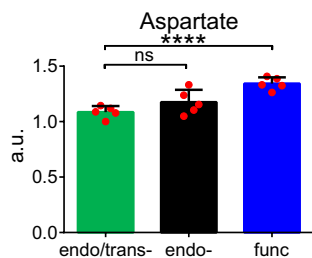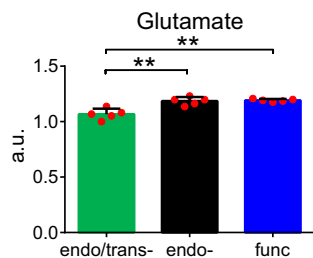

Supplement: S14 Fig — Concentration of ketogenic amino acids Trp, Ile, Leu, Lys, Asp and Glu in tubGS>mtEcoBI endo/trans- (UAS-mtHsdM.UAS-mtHsdS/+;UAS-mtHsdR K477R/tubGS), endo- (UAS-mtHsdM.UAS-mtHsdS/+;UAS-mtHsdR D298E/tubGS) and func (UAS-mtHsdM.UASmtHsdS/+;UAS-mtHsdR/tubGS) strains at day 6 after induction with 200 μM MP, ns–not significant, p<0.05 (*), p<0.01 (**), p<0.0001 (****), n = 5. (PDF) [file pgen.1008410.s018.pdf]

**A**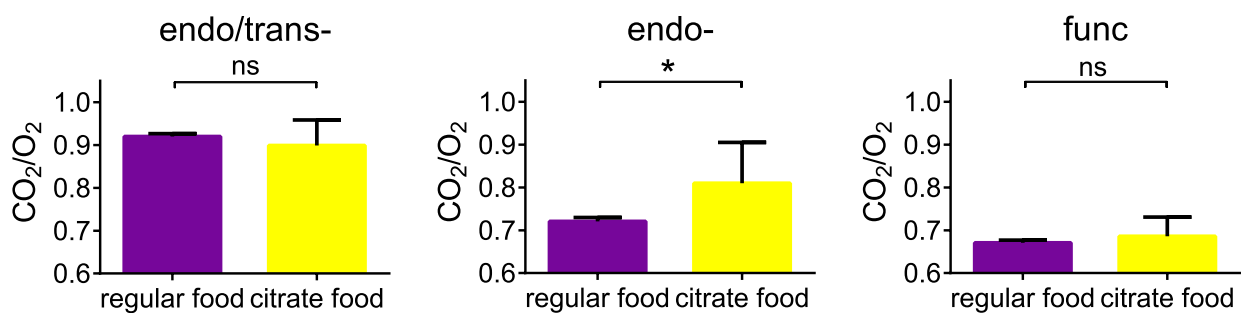**B**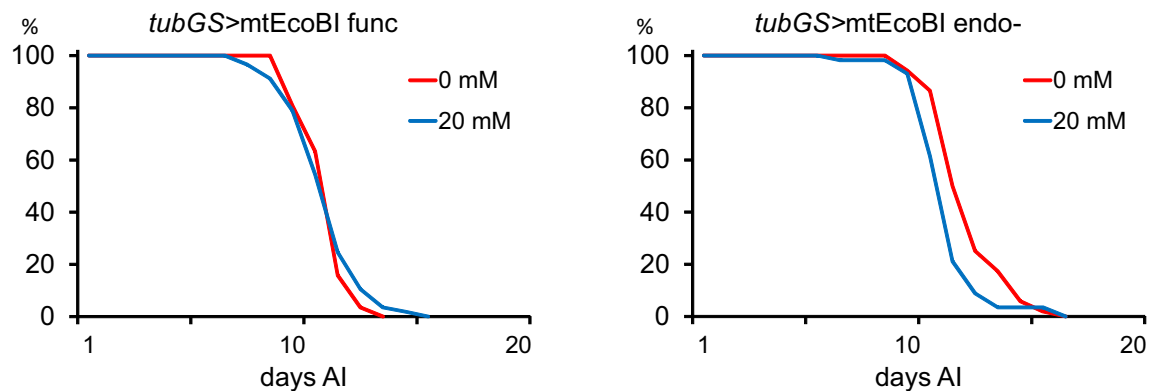**C**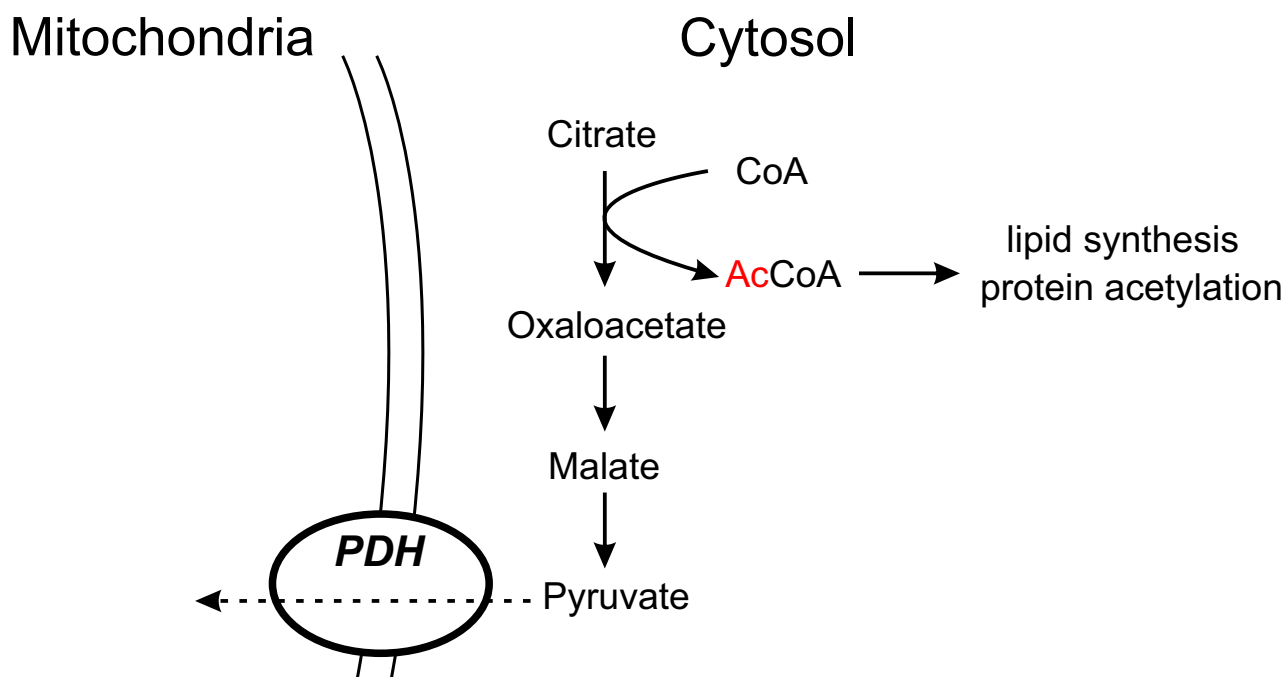

Supplement: S16 Fig — (A) Side-by-side comparison of RER (data from Figs 3E and 5G) of tubGS>mtEcoBI endo/trans- (UASmtHsdM.UAS-mtHsdS/+;UAS-mtHsdR K477R/tubGS), endo- (UAS-mtHsdM.UASmtHsdS/+;UAS-mtHsdR D298E/tubGS) and func (UAS-mtHsdM.UAS-mtHsdS/+;UASmtHsdR/tubGS) strains kept on regular food with 200 μM MP or citrate-supplemented food with 200 μM MP on day 6 after induction, ns-not significant, p<0.05 (*). (B) Lifespans tubGS>mtEcoBI endo/trans- (UAS-mtHsdM.UAS-mtHsdS/+;UAS-mtHsdR K477R/tubGS), endo- (UASmtHsdM.UAS-mtHsdS/+;UAS-mtHsdR D298E/tubGS) and func (UAS-mtHsdM.UASmtHsdS/+;UAS-mtHsdR/tubGS) strains on food containg 200 μM MP with or without 20 mM oxaloacetate. Lifespans on food without oxaloacetate are replicates from Fig 2A to provide a better comparison with oxaloacetate effect. (C) Schematic representation of conversion of cytosolic citrate to pyruvate, PDH—pyruvate dehydrogenase. (PDF) [file pgen.1008410.s020.pdf]
