# Supplementary material for: Manipulating mtDNA in vivo reprograms metabolism via novel response mechanisms
Source: PLoS Genet. 2019 Oct 4;15(10):e1008410. doi: 10.1371/journal.pgen.1008410 (PMC6795474; doi:10.1371/journal.pgen.1008410)
Supplement: S13 Fig — Levels of glycolysis and serine synthesis intermediates from tubGS>mtEcoBI (UAS-mtHsdM.UASmtHsdS/+;UAS-mtHsdR K477R/tubGS), endo- (UAS-mtHsdM.UAS-mtHsdS/+;UAS-mtHsdR D298E/tubGS) and func (UAS-mtHsdM.UAS-mtHsdS/+;UAS-mtHsdR/tubGS) strains 6 days after induction with 200 μM MP, ns–not significant, p<0.05(*), p<0,01 (**), n = 3–5. Dashed arrows represent more than one reaction between intermediates. (PDF) [file pgen.1008410.s017.pdf]

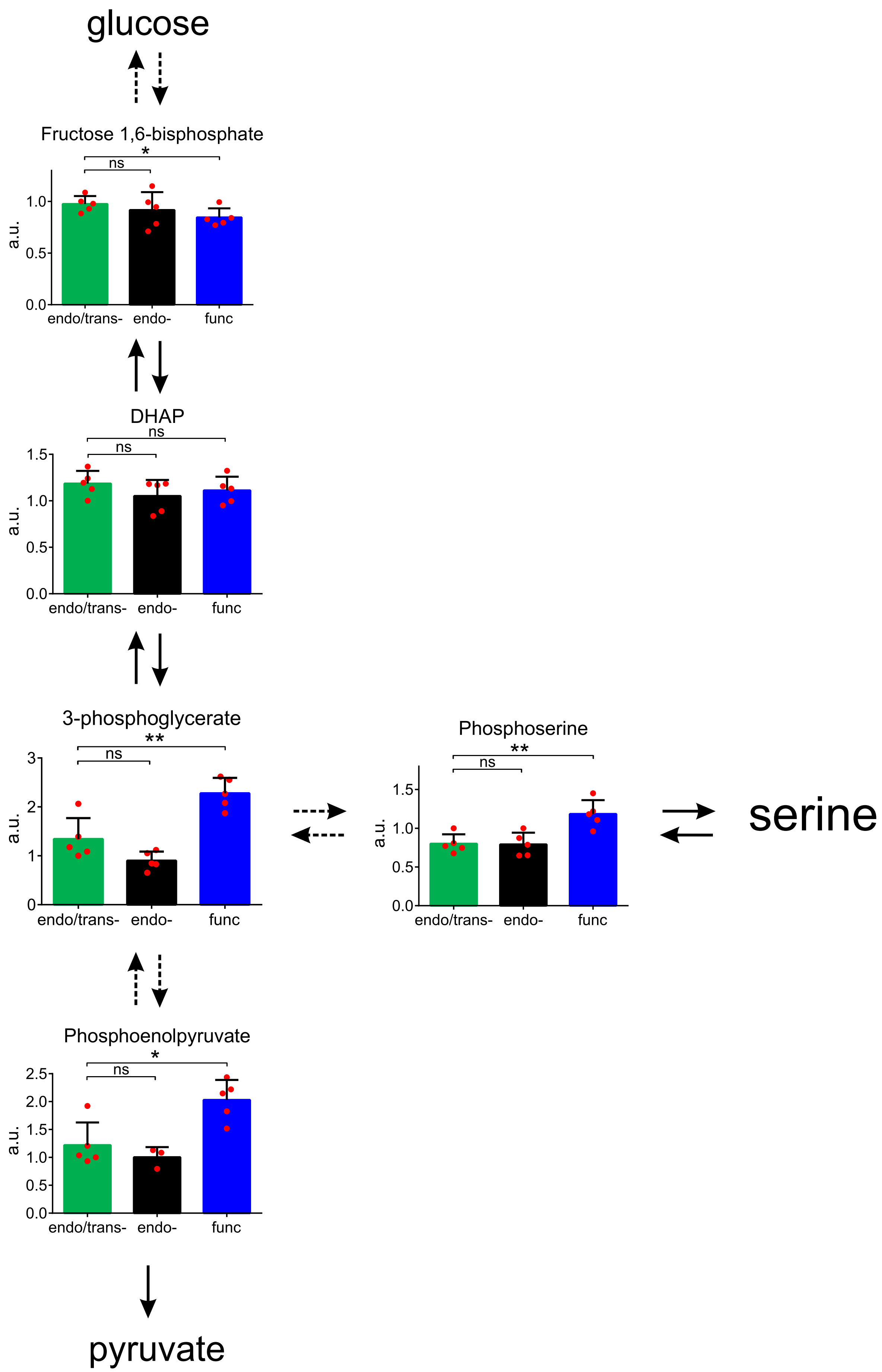
glucose
Fructose 1,6-bisphosphate
*
ns
a.u.
1.0
0.5
0.0
endo/trans-
endo-
func
DHAP
ns
ns
1.5
1.0
0.5
0.0
3-phosphoglycerate
**
ns
3
2
1
0
Phosphoserine
**
ns
serine
Phosphoenolpyruvate
*
ns
2.5
2.0
1.5
1.0
0.5
0.0
pyruvate
