# Supplementary material for: Manipulating mtDNA in vivo reprograms metabolism via novel response mechanisms
Source: PLoS Genet. 2019 Oct 4;15(10):e1008410. doi: 10.1371/journal.pgen.1008410 (PMC6795474; doi:10.1371/journal.pgen.1008410)
Supplement: S15 Fig — (A) Densitometry curve of Ponceau S—stained protein signal, data points are mean of two replicates, value represents R2. (B) Antibody- and Ponceau S-stained western membranes used in quantifications of global acetylation, H3 acetylation (rep1 and rep2 stand for separate technical repeats), mitochondrial acetylation and global PARylation signal from tubGS>mtEcoBI endo/trans- (UASmtHsdM.UAS-mtHsdS/+;UAS-mtHsdR K477R/tubGS), endo- (UAS-mtHsdM.UASmtHsdS/+;UAS-mtHsdR D298E/tubGS) and func (UAS-mtHsdM.UAS-mtHsdS/+;UASmtHsdR/tubGS) strains. Quantification areas are shown when less than a full lane was taken for quantifications due to nonspecific signal on acetylated lysine westerns caused by the vicinity of a protein run marker. AI—after induction with 200 μM MP. (C) Total NAD and NAD+/NADH ratio in tubGS>mtEcoBI endo/trans- (UAS-mtHsdM.UAS-mtHsdS/+;UAS-mtHsdR K477R/tubGS), endo- (UAS-mtHsdM.UAS-mtHsdS/+;UAS-mtHsdR D298E/tubGS) and func (UASmtHsdM.UAS-mtHsdS/+;UAS-mtHsdR/tubGS) strains 10 days after inducton with 200 μM MP, p<0.05 (*), p<0.0001 (****), n = 4–5. (PDF) [file pgen.1008410.s019.pdf]

A

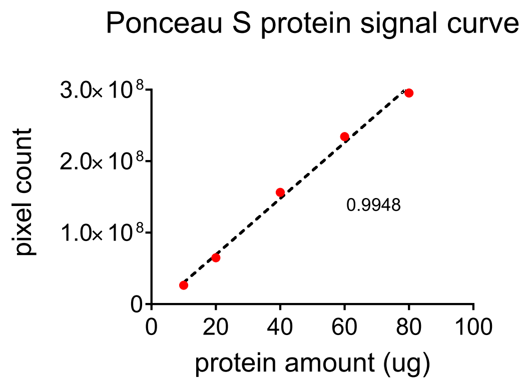
Ponceau S protein signal curve
pixel count
3.0x 10^8
2.0x 10^8
1.0x 10^8
0
0.9948
0
20
40
60
80
100
protein amount (ug)

B

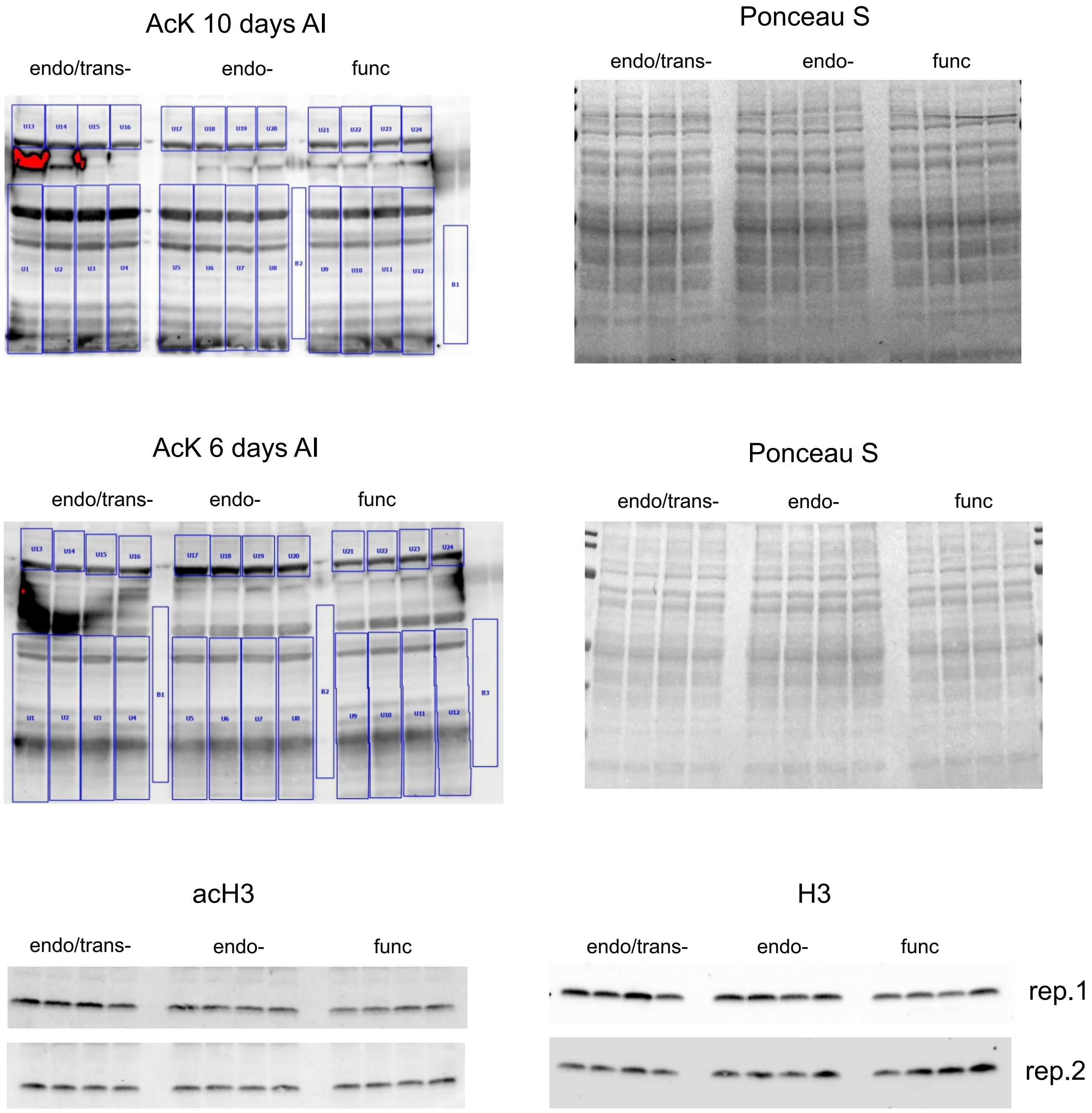
AcK 10 days AI
endo/trans-
endo-
func
Ponceau S
endo/trans-
endo-
func
AcK 6 days AI
endo/trans-
endo-
func
Ponceau S
endo/trans-
endo-
func
acH3
endo/trans-
endo-
func
H3
endo/trans-
endo-
func
rep.1
rep.2

AcK mito 10 days AI

endo/trans- endo- func

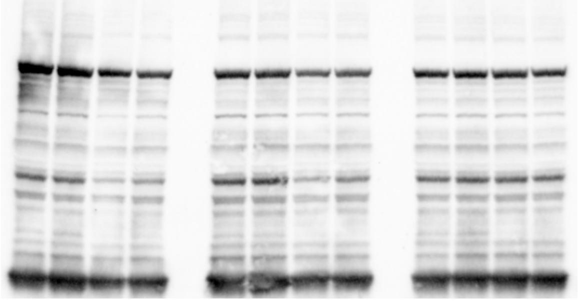

NDUFS3 mito 10 days AI

endo/trans- endo- func

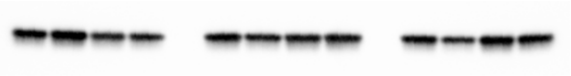

PARylation 6 days AI

endo/trans- endo- func

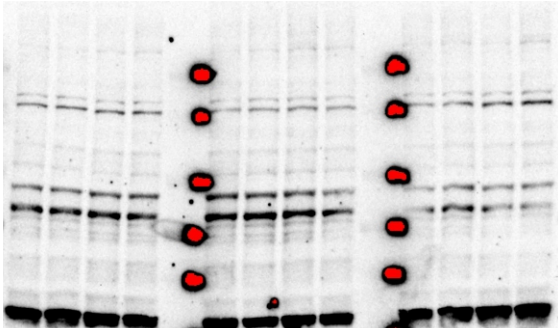

Ponceau S

endo/trans- endo- func

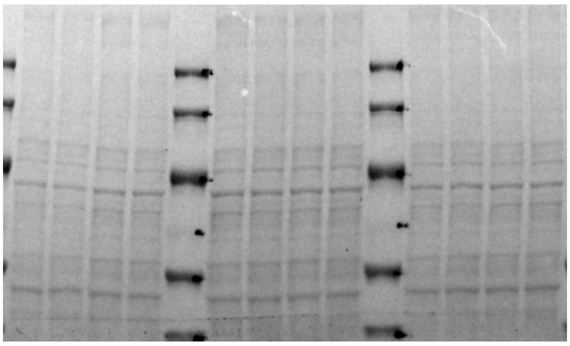

PARylation 10 days AI

endo/trans- endo- func

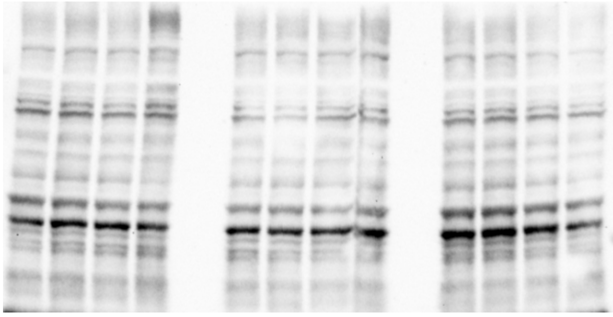

Ponceau S

endo/trans- endo- func

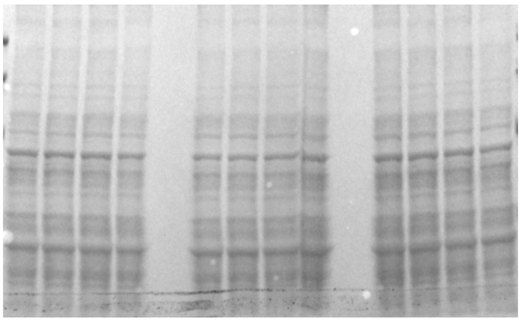

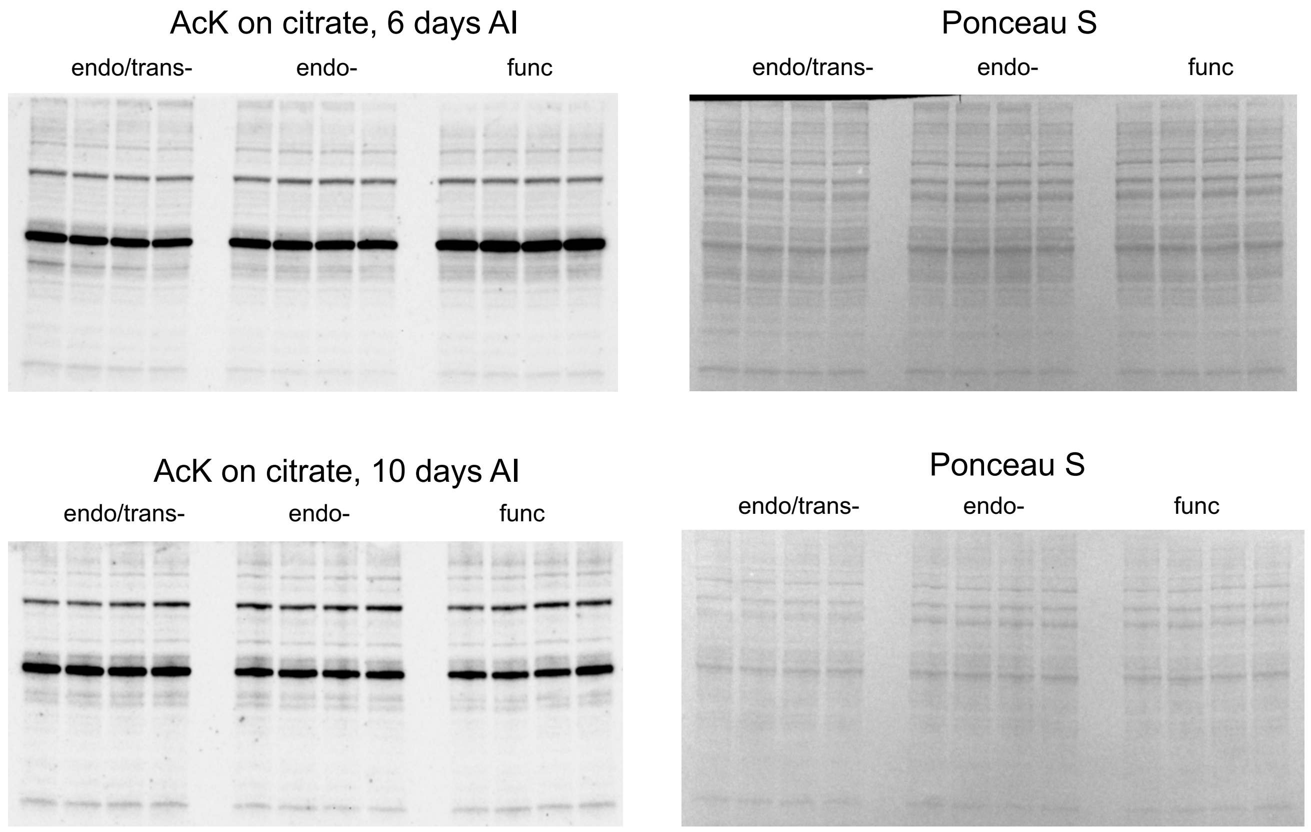
AcK on citrate, 6 days AI
endo/trans-
endo-
func
Ponceau S
endo/trans-
endo-
func
AcK on citrate, 10 days AI
endo/trans-
endo-
func
Ponceau S
endo/trans-
endo-
func

C

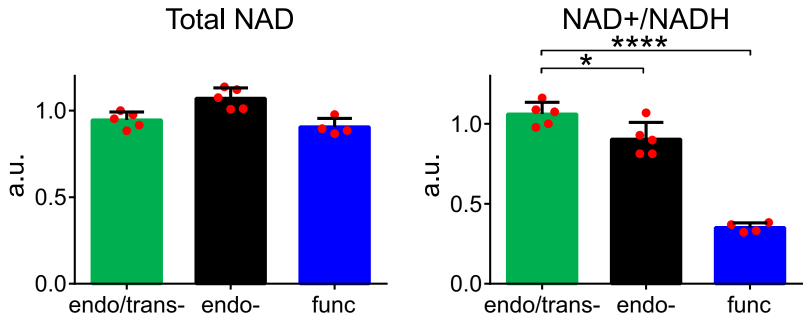
Total NAD
a.u.
1.0
0.5
0.0
endo/trans-
endo-
func
NAD+/NADH
****
*
a.u.
1.0
0.5
0.0
endo/trans-
endo-
func
